# Recent Progress on Semiconductor Heterojunction‐Based Photoanodes for Photoelectrochemical Water Splitting

**DOI:** 10.1002/smsc.202100112

**Published:** 2022-03-13

**Authors:** Shengnan Li, Weiwei Xu, Linxing Meng, Wei Tian, Liang Li

**Affiliations:** ^1^ School of Physical Science and Technology Jiangsu Key Laboratory of Thin Films Center for Energy Conversion Materials & Physics (CECMP) Soochow University Suzhou 215006 P. R. China

**Keywords:** charge carrier transfer, heterojunctions, photoanodes, photoelectrochemical (PEC) water splitting

## Abstract

For photoelectrochemical (PEC) water splitting, the utilization of semiconductor heterojunctions as building blocks for photoanodes allows for high light absorption, effective charge separation, and superior redox capability, greatly boosting the solar energy conversion efficiency. This review mainly focuses on the construction of heterojunction photoanodes, improvement strategies of carrier transmission, and their application in PEC water splitting. First, a series of carrier dynamics characterization methods are introduced to reveal the principle and significance of promoting carrier transport in heterojunctions. Then, from the perspective of the mechanism of promoting the separation and transport of charge carriers, several strategies are summarized and analyzed, including the micro/nanostructure, energy band structure, photothermal effect, piezoelectric effect, pyroelectric effect, ferroelectric effect, and intermediate layer. Finally, the challenges and opportunities for heterojunction photoanodes in PEC reactions are explained clearly, which points the way forward for the development of heterojunctions.

## Graphical abstract

This review mainly introduces recent advances on the construction of heterojunction photoanodes, improvement strategies of carrier transmission, and their application in photoelectrochemical (PEC) water splitting. Finally, a view of the future directions in this field is provided. This review has certain guidance and reference significance for research on heterojunction photoanodes.

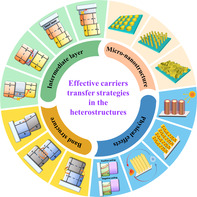

## 1 Introduction

Currently, the social demands for energy are increasing.^[^ ^1^ ^]^ Traditional fossil energy sources such as coal and oil are no longer sufficient to support current production and living needs.^[^ ^2^ ^]^ At the same time, the massive consumption of fossil fuels also causes considerable harm to the environment. Therefore, it is urgent to seek renewable clean energy to relieve the status quo of the energy crisis and environmental pollution.^[^ ^3^ ^]^ Hydrogen energy is considered one of the most promising green energy sources in the 21st century.^[^ ^4^ ^]^ Humans became interested in the application of hydrogen energy 200 years ago. The first single‐cylinder hydrogen internal combustion engine was invented in 1807.^[^ ^5^ ^]^ In 1938, the hypothesis of using hydrogen as aviation fuel was put forward, and it was first verified in practice in 1960. Subsequently, the first hydrogen fuel cell vehicle was released in 1972. Since the 1970s, in the face of environmental pollution and other crises, hydrogen energy research has been conducted extensively all around the world. There are currently a variety of hydrogen production technologies, dominated by the following three technical routes. The first is that fossil energy, including coal and natural gas, is reformed to produce hydrogen. The second is that hydrogen is produced from industrial byproduct gases such as those from coke ovens, the chlor‐alkali process, and propane dehydrogenation. The third technology is the production of hydrogen by water electrolysis.^[^ ^6^ ^]^ To achieve a low‐cost, pollution‐free, and simple hydrogen production method, solar energy photolysis of water for hydrogen production was developed.^[^ ^7^, ^8^ ^]^ However, the photocatalytic hydrogen production rate is extremely low due to the low light conversion efficiency.^[^ ^9^, ^10^ ^]^ Therefore, a compromise method is adopted between electrocatalysis and photocatalysis. Through the combination of solar energy and electric energy, the cost can be reduced, and the hydrogen production rate can be increased, making photoelectrochemical (PEC) water splitting favored by researchers.^[^ ^11^, ^12^, ^13^ ^]^ After generations of researchers sparing no exploration efforts, the field of PEC water splitting has harvested fruitful results.^[^ ^14^ ^]^

In 1972, Japanese scientists Fujishima and Honda first observed the phenomenon of photocatalytic water splitting on TiO_2_ photoanodes under ultraviolet irradiation.^[^ ^15^ ^]^ A typical PEC water splitting system consists of a photoanode, a photocathode, and an electrolyte. The photoanode is usually based on an n‐type semiconductor material with a light response. When excited by light, it can generate electron–hole pairs. In the case of an external circuit, the electrons generated in the photoanode flow to the photocathode, and hydrogen ions in the electrolyte accept electrons from the photocathode to produce hydrogen.^[^ ^16^ ^]^ The specific reaction process is as follows^[^ ^17^ ^]^

(1) $$\text{Photocathode}:2H^{+}+2e^{-}\to H_{2}$$

(2) $$\text{Photoanode}:2H_{2}O+4h^{+}\to O_{2}+4H^{+}$$

The total reaction equation of water splitting is

(3) $$2H_{2}O\to O_{2}+2H_{2}$$

The generation of PEC water splitting is a three‐step procedure: 1) Light absorption, in which the semiconductor is excited by light irradiation that is larger than the semiconductor bandgap generating electron and hole pairs. 2) The separation and recombination of the photogenerated electron and hole pairs in the semiconductor. 3) The occurrence of the surface redox reactions.^[^ ^18^, ^19^ ^]^ Light absorption can be adjusted by regulating the morphology, changing the sample thickness, and modulating the bandgap.^[^ ^20^, ^21^, ^22^, ^23^ ^]^ Regarding the separation and transport of electrons and holes, there are currently a series of solutions, such as element doping,^[^ ^24^, ^25^ ^]^ introducing defects,^[^ ^26^, ^27^ ^]^ and constructing heterojunctions.^[^ ^28^, ^29^, ^30^ ^]^ The oxygen evolution reaction (OER) on the photoanode surface involves a complex four‐electron four‐proton transfer process, and its kinetic process is very slow, which can generally be greatly alleviated by loading a cocatalyst.^[^ ^31^, ^32^, ^33^ ^]^ Among the above three influencing factors, the competition of separation and recombination between photogenerated electrons and holes is crucial for the PEC process. Appropriate amount of element doping and defects mainly change the conductivity and conductivity type of the sample to promote charge transfer.^[^ ^34^, ^35^ ^]^ According to the position where the introduced impurities and defects generate energy levels, they can be divided into shallow level and deep level. Shallow energy level means that a donor energy level closes to the conduction band or an acceptor energy level closes to the valence band (VB) is generated in the semiconductor, and basically all ionization is achieved at room temperature, thereby improving the conductivity of the semiconductor and changing the conductivity type.^[^ ^36^ ^]^ The deep energy level refers to the donor energy level far away from the conduction band and the acceptor energy level far away from the VB introduced in the semiconductor, which requires a large ionization energy, so there is no shallow energy‐level impurity that affects the carrier concentration and conductivity type. The deep energy‐level defects can act as effective recombination centers, reducing the carrier lifetime, known as a nonradiative recombination center, affecting the luminous efficiency, and increasing the resistivity of the material as a compensating impurity. Therefore, the type of defect states introduced is crucial for the performance of PEC water splitting.^[^ ^37^, ^38^ ^]^ The construction of heterojunctions meets the needs of ideal PEC photoanodes with a wide light absorption range, effective charge separation, and strong redox ability. Different semiconductor materials are combined to boost the effective separation and transport of electrons and holes assisted by the built‐in electric field at the interface. This review article takes several strategies by adjusting the band structure to promote carrier transport mainly from the point of view of constructing heterojunctions.^[^ ^39^ ^]^

This review first introduces the definition of heterojunctions and their advantages in acting as photoanodes for PEC water splitting. Second, several strategies for characterizing charge carrier dynamics are presented. Then, we specifically introduce micro–nanostructures, type‐II heterojunctions, Z‐scheme heterojunctions, gradient doping, various physical effects (photothermal, piezoelectric, pyroelectric, and ferroelectric effects), and the introduction of intermediate layers to enhance carrier transfer in heterojunctions. By applying the described carrier transport dynamics strategies to the heterojunctions, the carrier transport path will be further improved based on the intrinsic advantages of the heterojunctions, which will facilitate the photogenerated electrons and holes to reach their own reaction sites to take part in the reaction. Therefore, the review will have certain significance as a reference for future heterojunction modification work.

## 2 Definition of a Heterojunction

A heterojunction is the interfacial area at the contact point of two semiconductors. An ideal PEC photoelectrode should utilize as much of the sunlight as possible and have a strong redox potential, but the relationship between these two features is contradictory. A single semiconductor cannot meet these demands. A heterojunction is composed of different semiconductors.^[^ ^40^, ^41^, ^42^ ^]^ A wide bandgap semiconductor is selected to combine with a small bandgap semiconductor to absorb a wider range of light while it is expected to maintain a strong redox potential.^[^ ^43^, ^44^ ^]^ Heterojunctions more easily meet the requirements of wide light absorption and strong redox ability than a single semiconductor. Therefore, the construction of heterojunctions is quite extensive in the PEC field.^[^ ^45^ ^]^ According to the energy band alignment of the heterojunctions formed by the two semiconductors, they can be classified into straddling‐gap heterojunctions (type‐I), staggered‐gap heterojunctions (type‐II), and broken‐gap heterojunctions (type‐III).^[^ ^46^, ^47^ ^]^ In addition, there is a Z‐scheme heterojunction, and the charge transfer pathway exhibits a Z‐shape.^[^ ^48^, ^49^ ^]^ In light of the conductivity type of the semiconductor, heterojunctions can be divided into n–n junctions, p–p junctions, and p–n junctions.^[^ ^50^ ^]^

Generally, the construction of heterojunctions should meet the following conditions: similar crystal structures, lattice spacings, and thermal expansion coefficients. Constructing heterojunctions has been widely used as an effective means to promote carrier separation and transport. Heterojunctions have been widely developed to improve the PEC performance in photoelectric catalysis. In addition, the specific surface area of photoanodes may also be increased by constructing heterojunctions, which could enlarge the contact region between the photoanode and the electrolyte, strengthen light harvesting by compounding with a narrow gap semiconductor, and achieve performance improvements.^[^ ^51^ ^]^ However, constructing a heterojunction is not a perfect solution that is profitable and harmless. There are still unavoidable problems in the process of constructing heterojunctions, which are not conducive to carrier transmission and separation, thereby affecting the PEC performance.^[^ ^52^ ^]^ Due to the lattice mismatch of the two semiconductors forming heterojunctions, the bonding at the interface may not be tight enough or there may be a large number of defects, which is unfavorable for carrier transmission and separation at the interfaces of heterojunctions.^[^ ^53^ ^]^ At the same time, it is essential to take the energy band positions of the two semiconductors into account while constructing heterojunctions. The matched energy band structure forms a cliff‐like junction at the interface, which can bring high photocurrent and OER performance. However, with a poorly matched band structure, the interface barrier will form a spike‐like junction, which is not conducive to charge transfer. These problems need to be avoided and solved one by one to obtain a relatively perfect heterojunction. Therefore, it is necessary to provide relevant strategies for the carrier transmission problem of the heterojunction.

## 3 Carrier Dynamics Characterization Methods

There are three basic steps in a photocatalytic reaction: the semiconductor catalyst absorbs light larger than its bandgap producing photogenerated carriers, the carriers migrate to the catalyst surface, and the photogenerated carriers contact the reactants performing the catalytic conversion. The efficiency of converting light energy to chemical energy is equal to the product of the efficiencies of the following three processes: light capture, photogenerated charge separation and migration, and the high‐efficiency surface catalytic reaction. How to achieve efficient synergy of these three processes is a very critical scientific problem. First, light capture determines the theoretical efficiency of the maximum energy conversion of the catalyst. Second, the separation of photogenerated charges is a very complex process of multiple time scales and multiple space scales. The recombination process of the bulk, surface, and interface photogenerated carriers is inevitable mainly because the reactions involve excited states that occur in the femtosecond to millisecond time range. Therefore, it is necessary to study the dynamics of photogenerated carriers.^[^ ^54^ ^]^ In general, the characterization methods of the carrier transport dynamics are mainly based on the degree of recombination between photogenerated carrier, the transmission rate or the transmission efficiency and other parameters.

### 3.1 Time‐Resolved Photoluminescence Spectroscopy

Time‐resolved fluorescence analysis is known as transient fluorescence analysis. When the semiconductor material absorbs enough photons, the VB electron will be excited to a higher state, and the corresponding hole will remain in the VB, but the excited state electron will also move back to a lower energy level, and hole recombination and radiation emit fluorescence.^[^ ^55^ ^]^ In the short time after the material is excited instantaneously, the fluorescence intensity reaches its maximum value and then decays exponentially. The time in which the fluorescence intensity decays to 1/*e* of the initial time is defined as the fluorescence lifetime. Therefore, the time‐resolved fluorescence spectrum can be obtained through testing, and the fluorescence lifetime can be analyzed and fitted, thereby assisting the analysis of the lifetime of the excited electrons.^[^ ^56^ ^]^

At present, the pulse method for time‐resolved photoluminescence spectroscopy (TRPL) is the most commonly used technique, and time‐correlated single photon counting (TCSPC) is currently the main application technology of TRPL measurement. The principle is that the probability of detecting an emitted photon at a certain time *t* is proportional to the fluorescence intensity at that time point. Time resolution is an important parameter of fluorescence lifetime measuring instrument, which determines the shortest fluorescence lifetime that can be measured by the instrument, which is jointly determined by the excitation light source and the detector.

In the TCSPC, the choice of the excitation light source is very important, either various gas flash lamps or pulsed lasers can be used. The cost of the flash lamp is relatively low, the pulse given is basically in nanoseconds, and the pulse frequency is not high (10^4^–10^5^ Hz). Therefore, the data acquisition time is long, and the light intensity may drift during the measurement process, which affects the efficiency and accuracy of the measurement. However, pulsed lasers can give picosecond pulses, and the pulse frequency can be very high, but its price is relatively expensive. In terms of detectors, photomultiplier tubes (PMTs) and microchannel plate detectors (MCPs) are generally used. The latter has a faster response time and less interference. Theoretically, the instrument using pulsed laser and MCPs can detect the fluorescence lifetime of 10‐20 ps. Furthermore, the main features of TCSPC test are as follows: 1) Intuitive view of fluorescence decay curve. 2) High sensitivity, and low fluorescence intensity can be solved by prolonging the acquisition time. 3) The error of the random distribution of photons obeys the Poisson distribution, which is helpful for the data processing and judgment. 4) Direct and convenient for the recording of TRPL spectrum. 5) Deconvolution calculation is often required due to low measured fluorescence lifetime. This requires that the excitation signal of the instrument itself must be accurately measured, otherwise the calculated results will deviate greatly from the actual. 6) Complicated analysis of fluorescence lifetime from a mathematical point of view. 7) Data acquisition is time‐consuming.

### 3.2 Transient Absorption Spectrum

Transient absorption analysis refers to the material being excited from the ground state to the excited state by a beam of pumped light with high energy, and then the relaxation process of the excited state particles is detected by applying a beam of probe light with low energy, which can be used to evaluate carrier dynamics.^[^ ^57^ ^]^ In the ultrafast transient absorption analysis spectrum, the abscissa is the relative value of the probe light, and the ordinate *ΔA* represents the difference between the absorption spectra measured by the probe light irradiated on the sample before and after the addition of pumped light.^[^ ^58^ ^]^ Regarding the delay time of the pumped light, the resolution can reach picoseconds and even femtoseconds. The relaxation time is obtained by fitting the curve. The longer the relaxation time is, the longer is the electron lifetime and the better is the separation effect of photogenerated electrons and holes.

The detection light of nanosecond transient absorption spectrum (TAS) usually adopts a noncollimated “xenon lamp,” and the efficiency of detection light is low. It is necessary to increase the pump and xenon lamp power or use the μs pulse xenon lamp to achieve instantaneous high light intensity in order to obtain the TA signal. Furthermore, high‐energy pumping and high‐power detection also lead to solid samples that are easily ablated. In addition, the time resolution is mainly determined by the probe light and pump light pulse times. Currently, the pulse time of high‐power lasers is usually 5–10 ns, which limits the time resolution of TAS to 7–10 ns. However, pulsed white light laser as the detection light can greatly improve the detection efficiency. Moreover, the pulsed white light laser is a real collimated laser, and the detection spot is also smaller. For solid thin film samples, the scanning and moving sample holder can be used to achieve spatial resolution. In short, as the charge transfer or energy transfer in semiconductors is generally on the order of femtosecond and picosecond, TAS is one of the best methods to study the carrier relaxation process of semiconductors in real time.

### 3.3 Transient Photovoltage/Transient Photocurrent

Before the laser pulse with nanosecond width (5 ns) is emitted, the laser host transmits the pulse signal to the oscilloscope through the trigger signal line, and the oscilloscope is ready to measure and record the data after receiving the pulse signal: when the laser is irradiated on the photoanode absorbing laser photons, the free charges will rapidly drift and diffuse to the electrodes on both sides after a series of photoelectric conversion processes. At this time, if the photoanode and the external circuit form a loop, there will be a current response, and the magnitude of the current response is related to the directionally moving charges in the photoanode, as well as the impedance of the loop. The input impedance of the oscilloscope has two types: 50 ohm and 1 m ohm. When the input impedance of the oscilloscope is adjusted to 1 m ohm, the photoanode is equivalent to being connected in series with a large resistor, which is equivalent to an open circuit. This measurement method is generally called transient photovoltage (TPV) measurement. When the input impedance of the oscilloscope is adjusted to 50 ohm, the photoanode is equivalent to being connected in series with a small resistor, which is similar to a short circuit. This measurement method is generally called transient photocurrent (TPC) measurement.

The TPV spectrum provides kinetic information on the photogenerated charge separation of different samples. The photovoltage response includes two components: rising and decay. The rising part in TPV corresponds to the accumulation of electron concentration of the conductive substrate on the electrode (resembling capacitor charging). TPV decay refers to the recombination process of electrons leaving the conductive substrate (resembling capacitor discharge).

The working principle in the TPV is the Dember effect.^[^ ^59^ ^]^ When the semiconductor is exposed to light due to the diffusion length difference between photogenerated electrons and holes, a Dember photovoltage will be generated. At the same time, the light intensity in contact between the semiconductor surface and the body gradually weakens, so the diffusion rate of the photogenerated carriers, which changes from a high concentration to a low concentration, is also different. For this reason, the photogenerated electrons and holes partially drift to realize the establishment of a built‐in electric field. The stronger the light irradiates the semiconductor, the more photogenerated electrons are generated. When the stronger the light irradiates the semiconductor, more photogenerated electron–hole separation will be produced, and the higher the photovoltage will be.^[^ ^60^ ^]^ Similarly, when the light intensity remains the same, the high photovoltage represents the good separation capability of photogenerated electrons and holes. The decay process occurs when the light is removed, which is devoted to characterizing the recombination of the photogenerated electrons and holes in the heterojunctions.^[^ ^61^ ^]^ A long electron recombination lifetime indicates that the recombination rate is low. In contrast, a short electron recombination lifetime suggests that the recombination rate of the photogenerated electrons and holes is high.^[^ ^62^ ^]^

The TPC response measures the electronic transmission and collection in the semiconductors. The appearance of a positive spike under light illumination indicates that electrons accumulate on the surface of the semiconductor and that the photocurrent decays to a stable value, which means that the electrons accumulated on the surface are transferred to the electrolyte.^[^ ^63^ ^]^ A rapid decay certificates that there is a serious recombination of electrons and holes, and a high steady‐state photocurrent indicates excellent separation efficiency.^[^ ^64^ ^]^ If a negative spike appears after the light illumination is removed, it suggests that there is back electron and hole recombination.^[^ ^65^ ^]^

TPC/TPV can be used to study the kinetic information of photogenerated charge separation, including drift and diffusion processes during photogenerated charge separation. Drift processes generally refer to rapid separation processes that occur within particles while diffusion processes generally refer to charge transport between particles over a long period of time. In addition, by analyzing the shape, time scale, symbol, and intensity of the TPV spectrum, a series of information such as the speed, separation direction, separation mode, and separation degree of photogenerated charge separation and recombination can be obtained. TPC/TPV are effective means to study the behavior of photogenerated charges on the nanosecond time scale. They have the advantages of noncontact and lossless, and can directly reflect the kinetic information such as the separation direction, separation efficiency, and charge lifetime of photogenerated charges. For photocatalytic reactions, the separation and transport process of photogenerated electron–hole pairs can be analyzed by TPC/TPV, which can explore the mechanism of charge recombination and provide guidance for improving the high efficiency of charge separation.

### 3.4 Intensity Modulated Photocurrent/Photovoltage Spectroscopy

The intensity modulated photocurrent/photovoltage spectroscopy (IMPS/IMVS) measurement system mainly consists of three parts: 1) Light source system—xenon lamps, UV lamps, light‐emitting diodes (LBD), etc. are commonly used as light sources (dual light sources can be used, that is, one light source generates steady‐state incident light, and the other light source generates modulated light. A DC voltage can also be used to superimpose a modulated voltage to drive a light source at the same time, and the superposition of steady‐state light and modulated light can also be realized). 2) Light source drive system—the main equipment is a frequency response analyzer and a potentiostat, which generates a voltage or current signal to drive the light source to generate a modulated light signal. 3) Test and analysis system—the frequency response analyzer is used to compare and analyze the relationship between the amplitude and phase of the input signal and the output signal, and finally the test results are output by the microcomputer.

IMPS and IMVS are frequency domain detection technologies. The input signal is generally composed of a beam of stable background light and a beam of sinusoidally modulated light that has a small disturbance.^[^ ^66^ ^]^ The output signal is the corresponding steady‐state photocurrent with modulation. The IMPS and IMVS tests are similar to impedance tests.^[^ ^67^ ^]^ During impedance testing, a constant voltage or current signal is applied to the test system, and an alternate current (AC) signal with a certain amplitude is superimposed at the same time to control the frequency change. The frequency of the measured AC signal is consistent with the frequency of the applied AC signal, but the phase angle is offset, and the impedance value is related to the frequency that can be measured. In contrast, the light beam intensity shining on the photoelectrode changes rather than the amplitude of the voltage or current signal. The first quadrant of the IMPS represents the hole transfer and recombination process. The second, third, and fourth quadrants represent the diffusion of electrons and the response process of the resistance–capacitance circuit.^[^ ^68^ ^]^ The electron transmission time (*τ*_d_) and the electron diffusion coefficient (*D*_n_) can be calculated.^[^ ^69^, ^70^ ^]^ IMVS can be qualitatively analyzed: 1) Direct transition recombination between the conduction band (CB) electrons and VB holes inside the semiconductor.^[^ ^71^ ^]^ 2) Recombination through the surface states on the semiconductor surface, yielding the electron lifetime (*τ*_n_) and electron diffusion length (*L*_n_) that can be quantitatively calculated. Therefore, IMPS/IMVS can be used to characterize *τ*_n_, *L*_n_, and recombination degree in a heterojunction to obtain the transport properties of photogenerated electrons and holes in a heterojunction.

IMPS/IMVS is a measurement method in the frequency domain, which studies the system under test in a wide modulation frequency range and can distinguish dynamic processes with different speeds. At the same time, electrons and holes appear in different phases in opposite directions, and the dynamic process of electrons and holes can be distinguished. Therefore, IMPS/IMVS can obtain more dynamic microscopic process information than other conventional measurement methods.

## 4 Effective Strategies to Promote Carrier Transport in Photoelectrodes

To promote charge carrier transfer in heterojunctions, several effective strategies have been adopted, including constructing micro–nanostructures, modulating the energy band structure, utilizing pyroelectric, piezoelectric, pyroelectric, and ferroelectric effects, and introducing an intermediate layer.

### 4.1 Micro–Nanostructures

**Figure 1:**
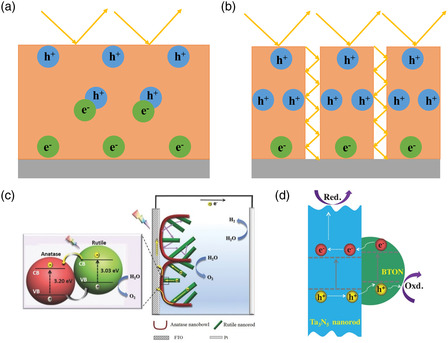
Depiction of the incident light processes, carrier transfer, and interfacial injection process of a) planar‐structured and b) nanorod arrays. c) Schematic for charge transport and light scattering in the TiO_2_ NR@NB array electrode. Reproduced with permission.^[^ ^80^ ^]^ Copyright 2016, Wiley‐VCH. d) Schematic for spatial charge transfer pathway. Reproduced with permission.^[^ ^81^ ^]^ Copyright 2019, Wiley‐VCH.

Heterojunctions with different micro–nanostructures exhibit different PEC performance characteristics. The following aspects determine the PEC performance of photoanodes: light absorption, bulk carrier separation, and surface charge carrier injection.^[^ ^29^, ^72^ ^]^ First, the most intuitive function of constructing a micro–nanostructure is to alter the specific surface area of the material, as shown in **Figure** **1**a,b. The larger the specific surface area is, the larger is the contact region between the photoanode and electrolyte, which would result in better PEC performance.^[^ ^73^ ^]^ Yang and co‐workers^[^ ^74^ ^]^ grew nanoporous BiVO_4_ coated onto SnO_2_ nanorods, which increased the contact area between BiVO_4_/SnO_2_ and electrolyte and was beneficial to the improvement of the performance of the photolysis of water and the rate of hydrogen production. Second, the construction of micro–nanostructures improves the light capture capabilities of photoanodes.^[^ ^73^ ^]^ Compared with conventional bulk planar structures, nanorods, nanosheets, nanobowl arrays, and other structured photoanodes exhibit better light refraction capabilities, which lengthens light traveling, extends the interaction between the light and the photoanode, and improves light harvesting efficiency. Tian et al.^[^ ^75^ ^]^ grew In_2_S_3_ nanosheets on WO_3_ nanowalls to enhance the light refraction and contact area with electrolytes compared to WO_3_ nanowalls, which caused WO_3_/In_2_S_3_ heterojunctions to possess PEC potential. Third, when the diffusion length is larger than the size of the material, holes can smoothly arrive at the photoanode/electrolyte interface and thus be involved in the oxidation reaction before hole recombination, which is conducive to PEC water splitting. The diffusion length is used to characterize the hole transport performance in the photoanode. For micro–nanostructured materials, the distance from photogenerated holes to the surface of the semiconductor/electrolyte is much less than that of bulk materials, which is beneficial to increase the probability of photogenerated minority carrier reaching the interface, inhibiting carrier recombination and improving PEC performance. Considering that BiVO_4_ has a short diffusion length of ≈70 nm, Pan et al.^[^ ^76^ ^]^ constructed a SnO_2_/TiO_2_/BiVO_4_ layered nanosheet@hollow microsphere array, and the thickness of electrodeposited BiVO_4_ was 20 nm. Because the thickness of BiVO_4_ is smaller than its hole diffusion length, the bulk separation of the SnO_2_/TiO_2_/BiVO_4_ photoanode was greatly improved, and this design helped the photogenerated holes reach the photoanode/electrolyte interface. Fourth, the width of the depletion layer has something to do with the characteristics of the material itself, along with the temperature and bias voltage. Therefore, a change in the morphology will change the width of the depletion layer. Compared with bulk materials, the proportion of depletion layers in nanomaterials is significantly increased, thereby promoting effective separation of the carriers. Cho et al.^[^ ^77^ ^]^ constructed CdS/CdO core–shell nanorods with different shell thicknesses and found that the shell thickness had a certain relationship with the width of the space charge region. Once the thickness of the CdO shell was shorter than the width of the depletion layer, 0.125 and 0.25 mM CdS/CdO photoanodes had a built‐in field within the thickness of the CdO shell. Shell thickness values of the 0.5 and 1 mM CdS/CdO photoanodes exceeded the width of the depletion layer, and there was a serious recombination phenomenon in the bulk. Fifth, when the thickness of the micro–nanostructure is within the range of the space charge layer, the photogenerated carriers can be effectively separated. If light absorption occurs in the deep bulk phase, there is insufficient driving force to separate the photogenerated electrons and holes.^[^ ^78^ ^]^ The micro–nanostructure better meets this requirement than the bulk structure, which allows for effective utilization of photogenerated charge carriers. In addition, the ordered micro–nanostructure provides an ordered channel for carrier transmission and inhibits bulk recombination.^[^ ^79^ ^]^ As shown in Figure 1c, the construction of a TiO_2_ nanorod/nanobowl array by Wang and co‐workers^[^ ^80^ ^]^ not only enhanced light absorption but also promoted the transport and separation of the photogenerated electrons along the rutile nanorods to the anatase nanobowls. Dong et al.^[^ ^81^ ^]^ constructed a 1D Ta_3_N_5_ nanorod/BaTaO_2_N (BTON) nanoparticle heterojunction, as shown in Figure 1d. The nanorod morphology promoted the directional transport of carriers, and 0D BTON nanoparticles had a short pathway for photogenerated holes to reach the surface, which was beneficial to the oxidation reaction of water splitting. **Table** **1** presents a summary of the performance of different micro–nanostructured heterojunctions.

**Table 1:** Summary of PEC performance for heterojunctions with micro–nanostructures

Photoanodes	Micro–nanostructures	*J* (1.23 *V*_RHE_)	*V*_onset_	Testing condition	H_2_ evolution rate	Ref.
NiFe/BiVO_4_/SnO_2_	Nanoporous/Nanorod	5.61 mA cm^−2^	/	1.0 m K‐B_i_ buffer	/	[74]
WO_3_/In_2_S_3_‐P	Nanowall/Nanosheet	1.61 mA cm^−2^	0.02 V_RHE_	0.1 m Na_2_SO_4_	/	[75]
SnO_2_/TiO_2_/BiVO_4_	Nanosheet@Microsphere	5.03 mA cm^−2^	/	0.5 m Na_2_SO_4_/0.1 m Na_2_SO_3_	/	[76]
		3.1 mA cm^−2^		0.5 m Na_2_SO_4_		
CdS/CdO	Core‐shell nanorods	4.35 mA cm^−2^ (0 V_SCE_)	‐1.40 V_SCE_	0.35 m Na_2_S/0.25 m Na_2_SO_3_	/	[77]
R‐TiO_2_/A‐TiO_2_	Nanorod@Nanobowl	1.24 mA cm^−2^	/	1 m NaOH	/	[80]
Ta_3_N_5_/BaTaO_2_N	Nanorod/Nanoparticle	/	/	/	27.3 μmol h^−1^	[81]
In_2_S_3_/F‐Fe_2_O_3_	Nanoparticle/Nanorod	2.21 mA cm^−2^	/	1 m KOH	/	[95]
FH‐TiO_2_	Nanosheet/Nanorod	2.24 mA cm^−2^	/	6 m KOH/10% v/v methanol	1.441 mmol g^−1^ h^−1^	[96]
					0.566 mmol g^−1^ h^−1^	
NFO/TiO_2_/ZFO/SnO_2_	Helix@Dendrite	1.0 mA cm^−2^	0.76 V_RHE_	1 m NaOH	/	[168]

The micro–nanostructures with different shapes increase the specific surface area, which are advantageous in light capturing and maximizing contacting area with the electrolyte. In addition, the ordered array structure provides a fast transport channel for photogenerated carriers. As micro–nanoengineering contributes to the light absorption, carrier transport, and separation capabilities of photoanodes, it is desirable to design and construct photoanodes with special shapes for water splitting.

### 4.2 Band Structure

Constructing a heterojunction is a universal and effective means to promote carrier separation. At present, type‐II and Z‐scheme heterojunctions have attracted the attention of researchers due to their advantages, and many studies have been carried out on them.^[^ ^82^, ^83^, ^84^ ^]^ However, it is not easy to select two types of semiconductors that are suitable for energy bands, so the search for a method to regulate and control the energy bands brooks no delay. Doping can adjust the energy band up or down by introducing donor impurities or acceptor impurities, and gradient doping is equivalent to constructing a continuous gradient energy band structure, which effectively solves the energy band problem.

#### 4.2.1 Type‐II Heterojunctions

**Figure 2:**
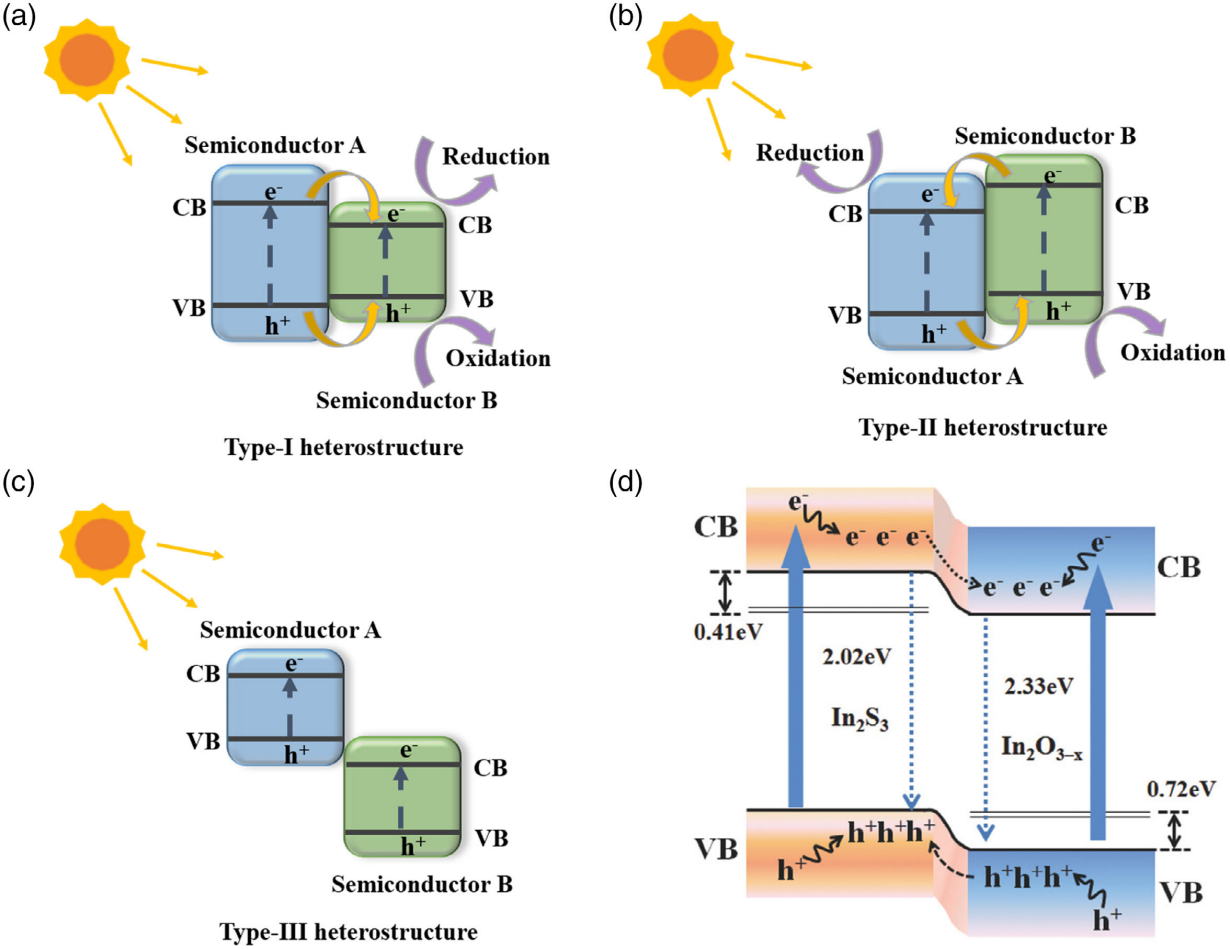
Illustration of the band alignment a) type‐I, b) type‐II, and c) type‐III. d) Schematic rendition of the In_2_O_3–*x*_/In_2_S_3_ heterostructure charge transfer tunneling mechanism. Reproduced with permission.^[^ ^100^ ^]^ Copyright 2018, Wiley‐VCH.

On the basis of band alignment of the two semiconductors, they can be classified into type‐I, type‐II, and type‐III heterojunctions.^[^ ^85^ ^]^ The structures of the three types of heterojunctions are shown in **Figure** **2**a–c. In the type‐I heterojunction, both electrons and holes are transferred from semiconductor A to semiconductor B. In this case, the photogenerated electron–hole pairs accumulate in semiconductor B and result in serious carrier recombination. In the type‐II heterojunction, the photogenerated electrons in semiconductor B are transported to semiconductor A, while the photogenerated holes on semiconductor A are transferred to semiconductor B, realizing spatial separation between photogenerated electron–hole pairs. In the type‐III heterojunction, there is no directional transport of photogenerated carriers between semiconductor A and semiconductor B. In addition, a narrow bandgap semiconductor is compounded on a wide bandgap semiconductor to construct a heterojunction, which reduces the bandgap of photoelectrodes, enhances the absorption of sunlight, and facilitates the extraction of photogenerated carriers. The advantage of efficient photogenerated electron–hole pairs separation makes constructing heterojunctions an effective way to improve carrier dynamics. In the past few decades, many researchers have discovered that a large number of type‐II heterojunctions can effectively separate electrons and holes and improve the performance of PEC water splitting, such as WO_3_/BiVO_4_,^[^ ^86^ ^]^ WO_3_/CdIn_2_S_4_,^[^ ^87^ ^]^ ZnO/ZnS,^[^ ^88^ ^]^ etc.^[^ ^89^, ^90^, ^91^ ^]^

As most heterojunctions cannot be formed at the same time, the preparation methods are mostly in a stepwise manner, that is, a semiconductor is first grown on a conductive substrate, and then another semiconductor is prepared.^[^ ^92^, ^93^, ^94^, ^95^ ^]^ For example, Gao et al.^[^ ^96^ ^]^ grew TiO_2_ nanorods on fluorine‐doped tin oxide (FTO) via a hydrothermal method, and the obtained samples were subjected to secondary hydrothermal treatment and then grew TiO_2_ ultrathin nanosheets in situ on TiO_2_ nanorods. The construction of this facet heterostructure promoted the efficiency of charge separation, prolonged the life of carriers, and was conducive to improving the performance of hydrogen production. Ye and co‐workers^[^ ^93^ ^]^ designed a B‐C_3_N_4_/Mo‐BiVO_4_ photoanode by three‐step processes, which increased the separation capability of photogenerated carriers at the interface of B‐C_3_N_4_ and Mo‐BiVO_4_.

The stepwise preparation of heterojunctions not only complicates the preparation process but is also likely to destroy the first prepared semiconductor during the preparation of the second semiconductor. Therefore, researchers are devoted to developing a one‐step process for fabricating heterojunctions.^[^ ^97^, ^98^ ^]^ For example, Cao et al.^[^ ^99^ ^]^ grew a 3D porous pyramidal In_2_O_3_/In_2_S_3_ heterojunction array by an ion exchange–induced synthesis strategy, obtaining the best performance at 1.23 V versus reversible hydrogen electrode (RHE) consisting of all In_2_S_3_ photoanodes and long water splitting stability. Hou and co‐workers^[^ ^100^ ^]^ synthesized a mesoporous In_2_O_3–*x*_/In_2_S_3_ 2D lateral heterostructure by in situ oxidation, as shown in Figure 2d, and the final performance was 21 times that of the In_2_S_3_ atomic layers. Dong et al.^[^ ^81^ ^]^ used a one‐step ammonia thermal route to prepare Ta_3_N_5_/BaTaO_2_N heterostructures and modulated ammonia thermal parameters for better PEC performance. Meng et al.^[^ ^101^ ^]^ built a CdIn_2_S_4_/In_2_S_3_ bulk heterojunction inside CdIn_2_S_4_ nanosheets by Ni‐phthalocyanine solution directly mixed with the CdIn_2_S_4_ precursor solution and achieved a separation efficiency of up to 90% at 1.23 V versus RHE.

The type‐II heterojunction promotes the separation and transport of carriers owing to the matched band structure and the built‐in electric field, but the type‐II heterojunction achieves this goal by sacrificing its redox capability. In addition, due to the existence of electrostatic interactions, photogenerated electron holes in the original photocatalyst will inhibit the interface transfer of photogenerated electrons and holes in other catalysts. Therefore, it is necessary to explore new types of heterojunctions to solve these drawbacks.

#### 4.2.2 Z‐Scheme Heterojunction

**Figure 3:**
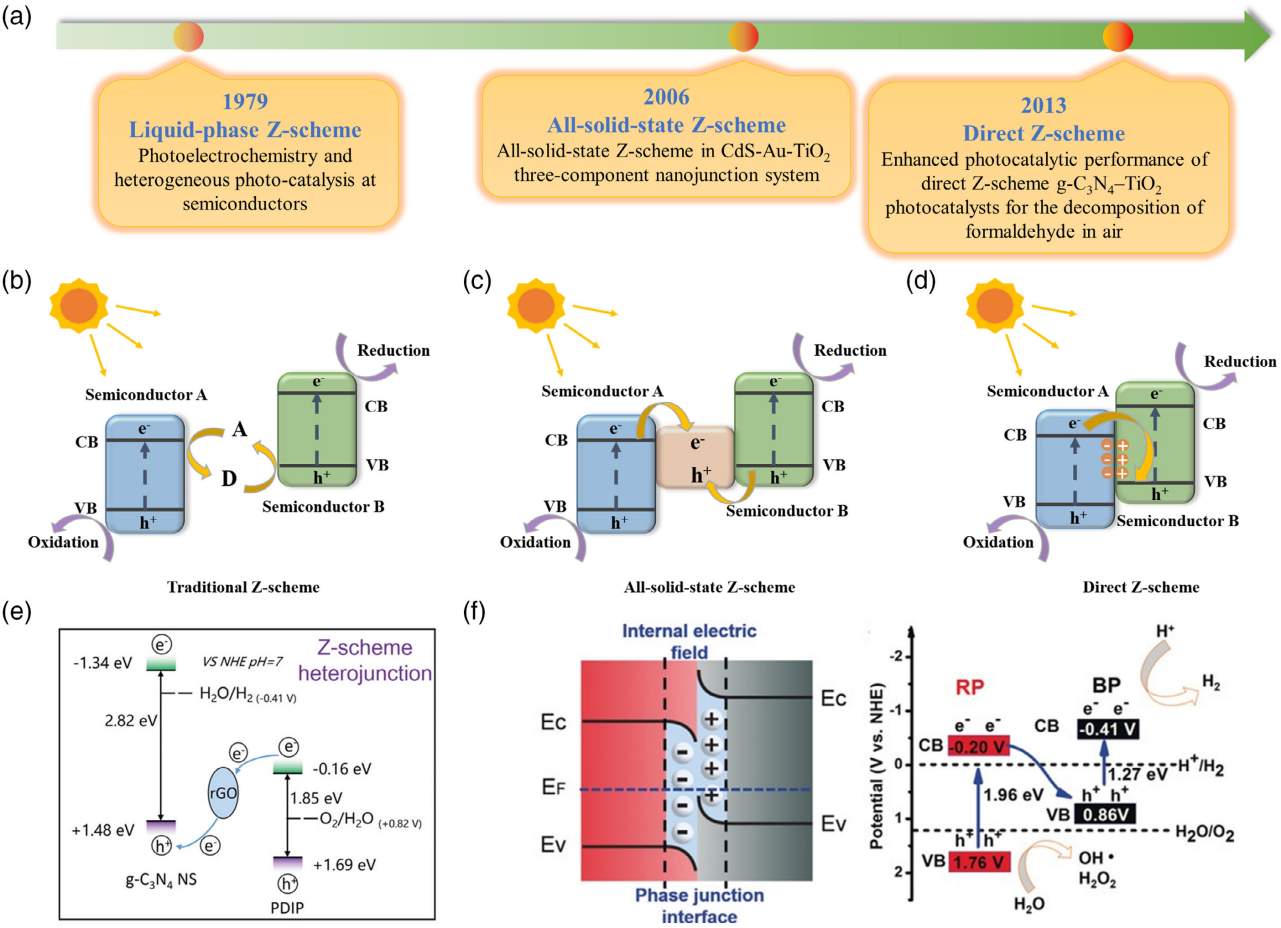
a) The development history of the Z‐scheme heterojunction. Illustrations of the band alignment, b) traditional Z‐scheme, c) all‐solid‐state Z‐scheme, and d) direct Z‐scheme. e) Transfer pathway of the g‐C_3_N_4_/rGO/PDIP Z‐scheme heterojunction. Reproduced with permission.^[^ ^105^ ^]^ Copyright 2021, Wiley‐VCH. f) Depiction of the band alignment and electron transport process in the BP/RP direct Z‐scheme heterojunction. Reproduced with permission.^[^ ^108^ ^]^ Copyright 2019, Wiley‐VCH.

To effectively separate electrons and holes, a type‐II heterojunction sacrifices the potential, which will adversely affect PEC water splitting. Z‐scheme heterojunctions with different carrier transfer pathways than type‐II heterojunctions have become a new choice for promoting photogenerated carrier separation. As the name implies, the Z‐scheme heterojunction refers to the charge transfer pathway in a “Z"‐shaped structure under illumination. Z‐scheme heterojunctions can be divided into three types: ionic Z‐scheme heterojunctions,^[^ ^102^ ^]^ all‐solid‐state Z‐scheme heterojunctions,^[^ ^103^ ^]^ and direct Z‐scheme heterojunctions.^[^ ^104^ ^]^ The development history and specific carrier transport mechanisms of the three Z‐scheme heterojunctions are shown in **Figure** **3**a–d.

The ionic Z‐scheme heterojunction first appeared in 1979. In Figure 3b, this heterojunction requires a redox electron mediator for charge transport, which speeds up the recombination rate of photogenerated electrons in the CB of semiconductor A and photogenerated holes in the VB of semiconductor B, promoting the efficient separation of photogenerated carriers. The commonly used redox electron mediators are [Co(bpy)_3_]^3+/2+^, Fe^3+^/Fe^2+^, IO^3−^/I^−^, NO^3−^/NO^2−^, and [Co(phen)_3_]^3+/2+^. In light of the limitation of the applicable environment of the ionic state Z‐scheme heterojunction, the all‐solid‐state Z‐scheme heterojunction has gradually replaced it. The all‐solid‐state Z‐scheme heterojunction is composed of a solid electronic conductor and two semiconductors, as shown in Figure 3c. The electronic conductor forms an ohmic contact with the two semiconductors, which promotes the recombination of photogenerated electrons in the CB of semiconductor A and photogenerated holes in the VB of semiconductor B. The commonly used electronic conductors are Au, Ag, Pt, Cu, Cd, W, and rGO. As shown in Figure 3e, Chen et al.^[^ ^105^ ^]^ successfully constructed a graphitic carbon nitride/rGO/perylene diimide polymer (g‐C_3_N_4_/rGO/PDIP) Z‐scheme heterojunction through a simple multistep method, which achieved high‐throughput charge transfer and effective photocatalytic water splitting. Through the TAS spectrum of g‐C_3_N_4_/rGO/PDIP, the average lifespan of the electrons trapped by PDIP was shortened, and the average lifetime of holes captured by PDIP was prolonged, which proved that charge transfer occurred in a timely and spatial manner in g‐C_3_N_4_/rGO/PDIP and successfully constructed a Z‐scheme heterojunction. Fu et al.^[^ ^106^ ^]^ built an α‐Fe_2_O_3_/Au/TiO_2_ ternary photoanode. By coating Au nanoparticles on the Fe_2_O_3_ surface, the photogenerated holes could be efficiently extracted. The presence of amorphous TiO_2_ on the surface accelerated the surface water oxidation reaction. Unfortunately, these mediators also generate reverse reactions and light‐shielding effects, which bring about a greatly reduced number of electron and hole pairs under light excitation. Furthermore, it is a rigorous problem for Z‐scheme heterostructures with electron mediators to maintain long‐term stability.

Inspired by nature, the direct Z‐scheme heterojunction mimics photosynthesis and is used to improve light conversion efficiency. As shown in Figure 3d, when two different semiconductors are in contact, the free photogenerated electrons in the CB of semiconductor A migrate to the CB of semiconductor B until the Fermi level reaches a balance. Accordingly, positive charges accumulate in semiconductor A, and negative charges gather in semiconductor B, forming a built‐in electric field. In the meantime, band edge bending occurs at the interface between semiconductor A and semiconductor B. Under light irradiation, the built‐in electric field accelerates the recombination of the free electrons in the CB of semiconductor A and free holes in the VB of semiconductor B. In the Z‐scheme heterojunction, an oxidation reaction of water splitting eventuates on the VB of semiconductor A, and the other half reaction of water splitting occurs on the CB of semiconductor B, thereby realizing the efficient detachment of photogenerated carriers. That is, the VB of semiconductor A with a relatively strong redox ability and the CB of semiconductor B with a comparatively strong redox ability are retained in the Z‐scheme heterojunction. As there is no mediator, the charges are directly transmitted through the interface, which shortens the transmission distance, benefits the occurrence of carrier recombination, and improves the photocatalytic potential of the photoelectrode. In addition, the direct Z‐scheme heterojunction avoids the occurrence of reverse reactions and light shielding phenomena, increases the density of effective photogenerated carriers, and enhances its photocorrosion resistance. The direct Z‐scheme black phosphorus/monolayer Bi_2_WO_6_ photoanode prepared by Hu and co‐workers^[^ ^107^ ^]^ showed strong photocatalytic performance in H_2_ production from water splitting and NO removal for air purification. In Figure 3f, Liu et al.^[^ ^108^ ^]^ designed and fabricated a black phosphorus/red phosphorus (BP/RP) heterophase junction, and the appropriate band structure with staggered alignment could help to possess the notable separation and transfer capacity of photogenerated electrons and holes. The evidence was provided by TAS via a direct Z‐scheme pathway. Zhou et al.^[^ ^61^ ^]^ used TPV decay to characterize the carrier transport of the photoanode. The carrier lifetime of ZnO/TiO_2_ was longer than that of TiO_2_, indicating that the construction of heterojunctions helped electron transport between the bulk phase and the interface. Xu et al.^[^ ^109^ ^]^ compared the TRPL of ZnIn_2_S_4_, ZnIn_2_S_4_/CdS, and ZnIn_2_S_4_/CdS/ZnO samples to prove that the successful construction of the Z‐scheme heterojunction promoted carrier separation and that the ZnO layer inhibited surface recombination and improved OER dynamics.

The current direct Z‐scheme heterojunction usually has two serious problems. 1) Due to the lack of mediators and the existence of weak van der Waals interlayer interactions, the recombination of photogenerated electrons in the CB of semiconductor A and photogenerated holes in the VB of semiconductor B is very low. For example, Wang et al.^[^ ^110^ ^]^ used two different ultrathin polymers, aza‐conjugated microporous polymers (CMP) and C_2_N nanosheets, to form a van der Waals Z‐scheme heterostructure. As the two polymers in the Z‐scheme heterojunction were combined through van der Waals forces, the carrier recombination at the interface increased, and the conversion efficiency of solar energy to hydrogen (STH) was 0.23%. Once RGO was introduced as a solid electronic mediator into the mental‐free Z‐scheme heterostructure, the STH value was further increased to 0.40%. 2) Cocatalysts and sacrificial agents are used to improve the surface catalytic ability beneficial to water redox reactions, specifically for the slow four‐electron involved OER. Wang and co‐workers^[^ ^110^ ^]^ adopted Pt and Co(OH)_2_ cocatalysts loaded onto the C_2_N releasing H_2_ and aza‐CMP nanosheets releasing O_2_, respectively, to further improve the photocatalytic performance.

Regardless of whether theoretically or experimentally, the straightforward characterization of direct Z‐scheme heterojunctions is still very challenging. Low et al.^[^ ^111^ ^]^ reported that in situ irradiated X‐ray photoelectron spectroscopy (ISI‐XPS) characterization, hydroxyl radical generation test, and density functional theory (DFT) simulation had been applied to confirm the formation of a direct Z‐scheme heterojunction obtained by compounding TiO_2_ with CdS. In ISI‐XPS, the Cd characteristic peaks underwent a negative shift, and the Ti characteristic peaks generated a positive shift, indicating that electrons were transferred from TiO_2_ to CdS via a Z‐scheme channel. In the hydroxyl radical test, as TiO_2_ was combined with OH^−^/H_2_O to generate hydroxyl groups but not CdS, hydroxyl groups were generated at the TiO_2_. Therefore, the detection of whether TiO_2_/CdS generated hydroxyl groups was used as a criterion for judging Z‐scheme heterojunctions. Wang et al.^[^ ^112^ ^]^ used electron spin resonance (ESR) and surface photovoltaic spectroscopy (SPV) to verify the Z‐scheme carrier transfer mechanism of S_v_‐ZnIn_2_S_4_/MoSe_2_ for photocatalytic hydrogen production. The SPV signal of S_v_‐ZnIn_2_S_4_/MoSe_2_ was lower than that of S_v_‐ZnIn_2_S_4_, indicating that the holes reaching the surface of S_v_‐ZnIn_2_S_4_/MoSe_2_ were reduced. Given that the CB of MoSe_2_ did not reduce O_2_ to ·O_2_^−^, the presence of ·O_2_^−^ signal in S_v_‐ZnIn_2_S_4_/MoSe_2_ was detected by ESR, which indicated that S_v_‐ZnIn_2_S_4_ accumulated a large number of photogenerated electrons, proving the formation of a Z‐scheme heterojunction.

Unlike the type‐II heterojunction that obtains effective bulk separation at the expense of redox capability, the Z‐scheme heterojunction ensures efficient separation of photogenerated electrons and holes while retaining a strong redox potential, which demonstrates the coexistence of high‐efficiency bulk phase separation and strong redox capability. Therefore, the direct Z‐scheme heterojunction has advantages in the field of PEC water splitting. However, reasonably designing and directly characterizing the premium direct Z‐scheme heterostructure are still challenging.

#### 4.2.3 Gradient Doping

The introduction of alien elements into the semiconductor is called doping. According to the semiconductor conductivity formula

(4) $$\sigma =n_{e}\mu_{e}+n_{h}\mu_{h}$$

*n* is the carrier concentration and *μ* is the carrier mobility.

If the semiconductor is doped with alien elements, the carrier concentration *n* increases, thereby increasing the conductivity of the semiconductor.^[^ ^113^ ^]^ In addition, the orbital hybridization of the introduced ions and semiconductors can change its electronic structure, CB, and VB. According to the doping positions, it can be divided into interstitial or substitutional doping. Substitutional doping needs to meet two conditions. The radius of the doped element and the element to be replaced are not very different, and the valence electron shell structures are similar. In line with the distribution of the doping amount, it can be divided into uniform doping and gradient doping. Uniform doping needs to consider the amount of doping. Otherwise, excessive uniform doping can easily form a recombination center, making the width of the depletion layer smaller and affecting the bulk separation.^[^ ^114^, ^115^ ^]^ Gradient doping not only has the advantages of uniform doping but can also form a gradient energy band structure, which can expand the width of the depletion layer and promote the bulk separation of carriers in the semiconductor. Hufnagel et al.^[^ ^116^ ^]^ proved, with the help of IMPS, that Sn doping only occurred on the hematite surface (surf‐Sn) and that the top 5 nm hematite layer (top‐Sn) showed higher charge transfer (*η*_ct_) efficiency than Sn doping that was evenly distributed throughout the whole hematite layer (bulk‐Sn) and the sample without Sn doping (undoped). Moreover, top‐Sn introduced a gradient band that facilitated the efficiency of charge transfer and separation, enhancing PEC performance. Meng et al.^[^ ^117^ ^]^ doped SnS_2_ nanosheets with In and Zn/In to form a gradient energy band, which increased the carrier concentration and improved the carrier separation efficiency.

**Figure 4:**
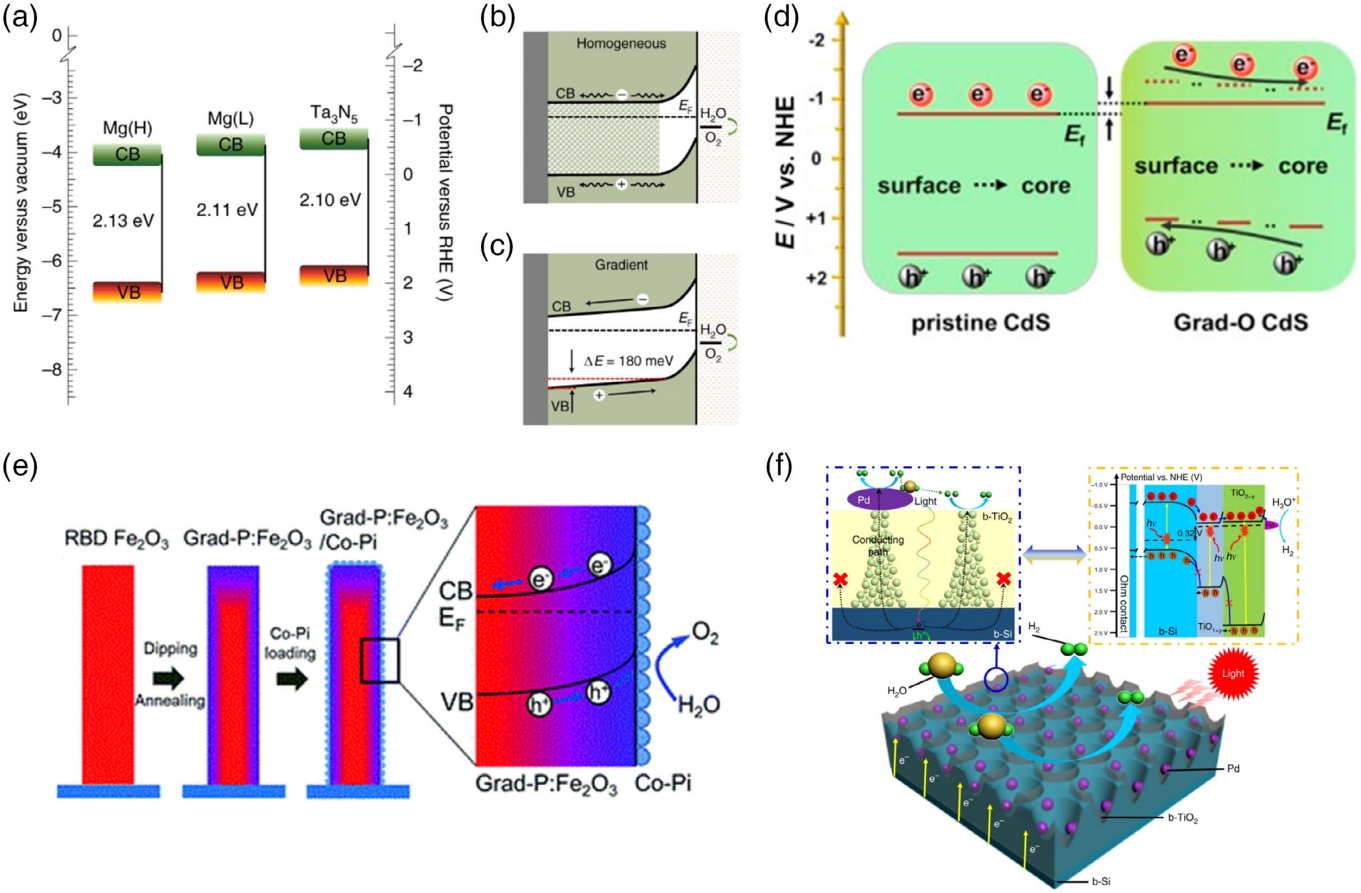
a) Schematic for pristine Ta_3_N_5_, Mg(L):Ta_3_N_5_, and Mg(H):Ta_3_N_5_. Schematics for band bending of b) homogenous and c) gradient Mg:Ta_3_N_5_. Reproduced with permission.^[^ ^121^ ^]^ Copyright 2020, Springer Nature. d) Schematic of pristine CdS and Grad‐O CdS sample charge transfer mechanisms. Reproduced with permission.^[^ ^122^ ^]^ Copyright 2018, Elsevier. e) Diagrams of the preparation process and band bending of a grad‐P: Fe_2_O_3_/Co‐Pi photoanode. Reproduced with permission.^[^ ^123^ ^]^ Copyright 2017, Royal Society of Chemistry. f) Schematic transfer tunneling and band alignment of the Pd nanoparticles/black TiO_2_/b‐Si photocathode for water reduction. Reproduced under the terms of the CC‐BY 4.0 license.^[^ ^127^ ^]^ Copyright 2018, The Authors, published by Springer Nature.

The types of doping elements can be divided into metal doping and nonmetal doping.^[^ ^118^, ^119^, ^120^ ^]^ The introduction of suitable metal ions into the semiconductor can generate a brand‐new energy level in the forbidden band to expand the response range of visible light and facilitate charge carrier separation. In **Figure** **4**a–c, Xiao and co‐workers^[^ ^121^ ^]^ showed that gradient Mg doping with the substitution of Ta^5+^ by Mg^2+^ in Ta_3_N_5_, which formed a gradient energy band and suppressed the carrier recombination caused by the defect states, thereby improving the charge separation efficiency, prolonged lifetimes as evidenced by TRPL spectroscopy, when compared to homogeneous Mg doping. Zhang et al.^[^ ^35^ ^]^ reported that linear electron energy loss spectroscopy (EELS) scanning implied that the Ta doped in hematite lattices must be in the form of substitution, and gradient Ta doping of the core–shell structure of hematite homojunction nanorods enhanced the photogenerated current density and reduced the turn‐on voltage. Nonmetal doping does not form acceptor or donor energy levels in the forbidden band, and it is not easy to form recombination centers. Instead, it narrows the forbidden band by shifting the top of the VB of semiconductor photocatalysts. Therefore, it has a wider range of solar spectral response. Our group^[^ ^75^ ^]^ produced S vacancies and gradient oxygen doping in In_2_S_3_ by PVP treatment, which not only increased the charge carrier concentration and reactive sites but also formed a gradient band structure that promoted the transfer and separation of carriers. Yu et al.^[^ ^122^ ^]^ doped 3D CdS dendritic nanorods with oxygen and distributed them along the radial gradient of the nanorods so that a continuous band‐bending structure was formed in the nanorods, which promoted the migration of photogenerated electrons from the surface to the core and Ti substrate, as illustrated in Figure 4d. Luo et al.^[^ ^123^ ^]^ constructed gradient P doping to increase the conductivity of the Fe_2_O_3_ nanoarray photoanode and cause upward bending of the energy band in a wider area (Figure 4e), thereby promoting charge separation along the radial direction of the hematite nanobeam.

Similar to doping, introducing vacancies can destroy the periodic arrangement of crystals and produce lattice distortions, which cause defect states to be generated in the bandgap, expand the range of light response, and promote the separation of photogenerated charge carriers.^[^ ^124^, ^125^, ^126^ ^]^ As depicted in Figure 4f, Zheng et al.^[^ ^127^ ^]^ used a simple method to construct a crystalline TiO_2_ protective layer with gradient oxygen defects on a silicon‐based photocathode. Conductive atomic force microscopy (C‐AFM) verified that the concentration of oxygen defects determined the channels by which the current passed through the TiO_2_ layer and affected the distribution and number of transport channels. Gradient oxygen defects were the major factors promoting and enhancing the PEC performance of the photovoltaic device.

Gradient doping can not only improve the conductivity and charge carrier concentration but also avoid the serious recombination problems caused by uniform heavy doping through gradient modulation of the doping concentration. In addition, a gradient energy band structure is constructed to widen the depletion layer and promote effective directional transport and separation of carriers.

**Table** **2** shows the performance comparison of the above three tuned heterojunction energy bands.

**Table 2:** Summary of PEC performance for heterojunctions with different energy band structures

Sample	Energy band structure	*J* (1.23 V_RHE_)	*V*_onset_	Testing condition	H_2_ evolution rate	Ref.
FH‐TiO_2_	Type‐II	2.24 mA cm^−2^	/	6 m KOH/10% v/v methanol	1.441 mmol g^−1^ h^−1^	[96]
					0.566 mmol g^−1^ h^−1^	
NiFeO_*x*_/B‐C_3_N_4_/Mo‐BiVO_4_	Type‐II	5.93 ± 0.3 mA cm^−2^	0.34 V_RHE_	PPB (pH = 7)	77.5 μM h^−1^	[93]
In_2_O_3_/In_2_S_3_	Type‐II	8.2 mA cm^−2^	0.02 V_RHE_	0.25 m Na_2_S/0.35 m Na_2_SO_3_	/	[99]
In_2_O_3–*x*_/In_2_S_3_	Type‐II	1.28 mA cm^−2^	0.75 V_RHE_	1.0 m KOH	/	[100]
Ta_3_N_5_/BaTaO_2_N	Type‐II	/	/	/	27.3 μmol h^−1^	[81]
CdIn_2_S_4_/In_2_S_3_	Type‐II	2.98 mA cm^−2^	0.02 V_RHE_	0.5 m Na_2_SO_4_	/	[101]
g‐C_3_N_4_/rGO/PDIP	Z‐scheme	/	/	/	15.80 μmol h^−1^	[105]
α‐Fe_2_O_3_/Au/TiO_2_	Z‐scheme	1.05 mA cm^−2^	/	1 m NaOH	18.67 μmol cm^−2^ h^−1^	[106]
BP/MBWO	Z‐scheme	/	/	/	21 042 μmol g^−1^	[107]
BP/RP	Z‐scheme	/	/	/	0.33 mmol g^−1^ h^−1^	[108]
BN ZnO/TiO_2_	Z‐scheme	2.75 mA cm^−2^	/	0.2 m Na_2_SO_4_	45.6 μmol cm^−2^ h^−1^	[61]
ZnIn_2_S_4_/CdS/ZnO	Z‐scheme	3.48 mA cm^−2^	− 0.03 V_RHE_	0.5 m Na_2_SO_4_	/	[109]
aza‐CMP/C_2_N	Z‐scheme	/	/	/	5.0 μmol g^−1^	[110]
aza‐CMP/RGO/C_2_N	Z‐scheme	/	/	/	10.0 μmol g^−1^	[110]
TiO_2_/CdS	Z‐scheme	/	/	/	11.9 mmol h^−1^ m^−2^ for CH_4_	[111]
S_v_‐ZnIn_2_S_4_/MoSe_2_	Z‐scheme	/	/	/	63.21 mmol g^−1^ h^−1^	[112]
Zn/In:SnS_2_	Gradient band structure	0.23 mA cm^−2^	0.3 V_RHE_	0.5 m Na_2_SO_4_	/	[117]
NiCoFe‐B_i_/Mg:Ta_3_N_5_/Nb	Gradient band structure	8.5 mA cm^−2^	0.40 V_RHE_	1.0 m KOH	/	[121]
NiFe(OH)_*x*_/Ta:Fe_2_O_3_@Fe_2_O_3_	Gradient band structure	3.22 mA cm^−2^	0.55 V_RHE_	1.0 m KOH	/	[35]
WO_3_/In_2_S_3_‐P	Gradient band structure	1.61 mA cm^−2^	0.02 V_RHE_	0.1 m Na_2_SO_4_	/	[75]
Grad‐O CdS/Ti	Gradient band structure	6.0 ± 0.1 mA cm^−2^	/	1.0 m Na_2_SO_4_/1.0 m N_2_H_4_·H_2_O	/	[122]
grad‐P:Fe_2_O_3_/Co‐Pi	Gradient band structure	2.0 mA cm^−2^	/	1 m KOH	/	[123]
Pd/b2‐TiO_2_/b‐Si	Gradient band structure	8.3 mA cm^−2^ (0 V_RHE_)	0.32 V_RHE_	1.0 m NaOH	/	[127]

### 4.3 Regulating Heterojunction Photoelectrodes with Photothermal, Piezoelectric, Pyroelectric, and Ferroelectric Effects

Although type‐II and Z‐scheme heterojunctions are extensively used, we can use semiconductors with special properties, such as photothermal and electrical properties, to further enhance the separation and transport of carriers in the heterojunctions.^[^ ^128^ ^]^ The photothermal effect mainly reduces the reaction barrier and accelerates carrier transport through in situ heating. The piezoelectric effect, pyroelectric effect, and ferroelectric effect enhance the performance of PEC water splitting by enhancing the built‐in electric field of the heterojunction.

#### 4.3.1 Photothermal Effect

Sunlight is an electromagnetic wave. According to the thermal effect of the electromagnetic wave, the inside of a material is neutral and has two types of charges with the same amount of electricity. The alternating electric field in the propagation of electromagnetic waves makes the positive and negative charges move in the material. This process is accompanied by collision and friction, showing a “hot” phenomenon. As the frequency of infrared rays is close to the frequency of the material, it is easier to cause resonance of the material, and the thermal effect is significant. For example, Zhang et al.^[^ ^129^ ^]^ used the photothermal effect of Cu_2_S to enhance the light absorption of visible light and near‐infrared (NIR) light radiation and to profitably increase the rate of OER due to the in situ heating of Cu_2_S.

**Figure 5:**
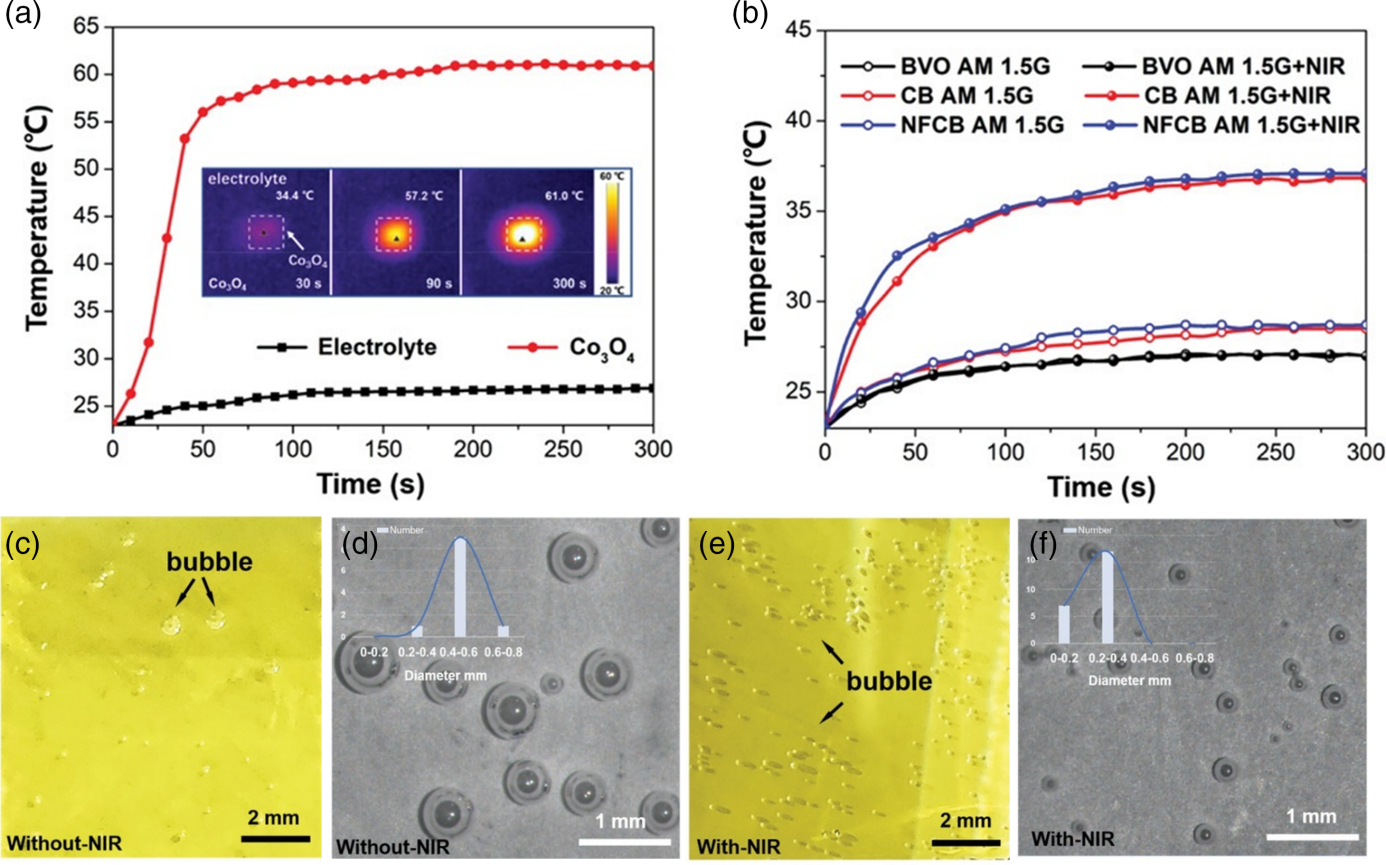
a) Temperature changes of the electrolyte solution and Co_3_O_4_ with time. b) Temperature–time graphs of BVO, CB, and NFCB photoanodes under AM1.5G illumination with or without simultaneous NIR light irradiation. Digital images of NFCB composite photoanode. O_2_ evolution process c,d) without e,f) with NIR light irradiation under the same current density. Reproduced with permission.^[^ ^131^ ^]^ Copyright 2021, Wiley‐VCH.

Generally, there are a number of photothermal materials: organic composites, noble metals, carbon‐based materials, and transition metal semiconductors. The photothermal effect is mostly based on the principle of surface plasmon resonance. The electromagnetic field of the surface plasmon is localized in a range far smaller than the wavelength scale, and the energy of the incident light is concentrated in the deep subwavelength or even nanometer‐scale spatial range through the surface plasmon, which makes the energy density of the light and local electric field strength enhanced, forming so‐called “hot spots,” indicating that surface plasmons can significantly enhance the interaction of light and matter. The characteristics of surface plasmon polaritons are closely related to materials, morphology and structure. When the resonance wavelength of surface plasmon polaritons is close to the wavelength of incident light, the photothermal effect is the strongest and the photothermal conversion efficiency is the highest. Moreover, the free electron concentration, mobility, and interband/intraband transition of carriers are three important factors that affect the properties of surface plasmons. Hot carriers will be generated under light excitation and can excite the electronic energy level or vibrational state of the surface plasmon or adsorbed molecules before generating heat, making them in a more active excited state, thereby greatly speeding up the chemical reaction rate. The electrons or holes are resonated by light shining on the semiconductor, resulting in nanoscale local heating.^[^ ^130^ ^]^ Introducing the photothermal effect into the catalytic process has two obvious advantages. First, a catalyst with a photothermal material can realize self‐heating under visual or NIR light without an external heating device. Second, heat is restricted to the surface of the catalyst owing to local heating, eliminating the influence of electrolyte temperature. When the photothermal materials are irradiated by NIR light, the photoanodes are heated in situ, and the increase in temperature causes the thermal movement of molecules in the material to increase significantly, thereby promoting electron transmission, accelerating reaction kinetics, and promoting gas escape. He et al.^[^ ^131^ ^]^ showed that NiOOH/FeOOH/Co_3_O_4_/BiVO_4_ combined standard sunlight and NIR irradiation to expand light absorption, and in situ heating promoted charge transfer while increasing the water oxidation kinetics, as shown in **Figure** **5**. At the same time, they also proved that the photothermal effect has universal significance for the improvement of photoanode performance. In addition, the measured temperature is much lower than the actual temperature. Increasing the temperature in situ in the nanoscale region can reduce the reaction barrier and promote the photocatalytic water splitting reaction. Zhang et al.^[^ ^132^ ^]^ showed that Ni_3_S_2_ was heated in situ with the assistance of the photothermal effect and provided evidence that the reaction potential decreased as the luminescence time increased. Jin and co‐workers^[^ ^133^ ^]^ proved that the temperature increase of the Co_3_O_4_ electrode caused by NIR irradiation promoted the electrolyte to penetrate into the photoelectrode, and greater amounts of active material were exposed to the electrolyte, which may be due to the large electrochemical active surface areas (ECSAs).

In short, the advantages of using photothermal materials to construct heterojunctions are significant. On the basis of the heterojunction itself, the addition of the photothermal effect brings about a widening of the light absorption range, speeding up charge transport, improving OER reaction kinetics and other advantages, and realizing a further improvement in the performance of the heterojunction.

#### 4.3.2 Piezoelectric Effect

**Figure 6:**
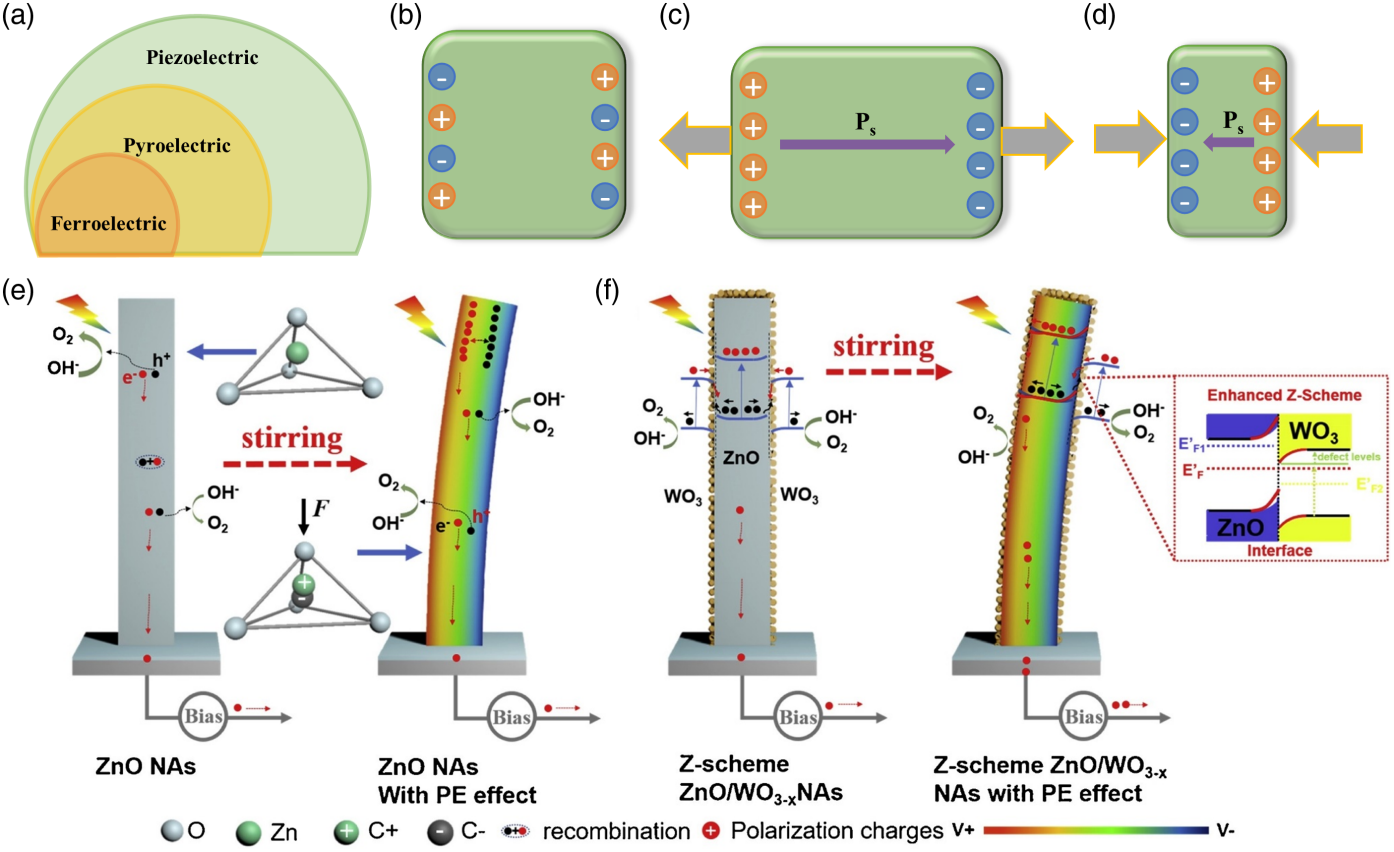
a) The relationship between piezoelectric materials, pyroelectric materials, and ferroelectric materials. Piezoelectric semiconductors b) without strain, c) under tensile stress, and d) under compressive stress. e,f) Schematic band bending diagrams of ZnO and ZnO/WO_3–*x*_ with the piezoelectric effect. Reproduced with permission.^[^ ^138^ ^]^ Copyright 2019, Elsevier.

According to the structural symmetry, the crystal structure can be divided into 7 crystal systems and 32 point groups, 20 of which do not have center symmetry, and their electric dipole moments can be changed due to elastic deformation, so they have piezoelectric such as ZnO, CdS, C_3_N_4_, MoS_2_, MoSe_2_, GaN, InGaN, ABO_3_, etc. Ten point groups with a unique polar axis in a piezoelectric body can exhibit spontaneous polarization, that is, electric polarization exists even in the absence of an electric field. They generate electric charges due to heating, so they are called pyroelectrics including ZnO, CdS, Bi_2_S_3_, ABO_3_, etc. Among these polar crystals, the crystal whose spontaneous polarization direction is changed by applying an external voltage is a ferroelectric, for example, ABO_3_. In addition to conventional oxides and sulfides that can be used as photoanode, the flexible choice of ABO_3_ structure of A and B cations makes the energy band structure tunable, making it one of the choices for photoanode. Piezoelectric materials have a noncentrosymmetric crystal structure. Due to their specific crystal structure, the electric dipoles generated in the material body change with the strain of the material.^[^ ^134^ ^]^ Generally, piezoelectric materials can be divided into three types of materials: ferroelectric materials, pyroelectric materials, and piezoelectric materials, and their relationships are shown in **Figure** **6**a.^[^ ^135^ ^]^ The introduction of pyroelectric materials and ferroelectric materials is described in Section 4.3.3 and 4.3.4, respectively.

Piezoelectric materials will not have a polarization electric field under any external force. Once a tensile stress is applied to the piezoelectric material, a polarization field emerges inside the piezoelectric material, and the orientation of the polarization field for applying compressive stress is the opposite. The specific schematic diagrams are shown in Figure 6b–d.^[^ ^136^ ^]^

The presence of the piezoelectric effect redistributes the charges at the interface of the heterojunction and changes its built‐in field and space charge region. Zhang and co‐workers^[^ ^137^ ^]^ constructed a Pt/ZnO/Co‐Pi photoanode, in which the piezoelectric effect of ZnO under the action of mechanical energy effectively enhanced the electric field intensity, prevented photogenerated charges from recombining, and enhanced the water splitting performance of Pt/ZnO/Co‐Pi. Therefore, building a heterojunction in PEC will change the interface band bending, increase the strength of the built‐in field, and expand the depletion layer by drawing on the support of the piezoelectric potential in the piezoelectric material, which further promotes the separation and transport of electrons and holes. Chen et al.^[^ ^138^ ^]^ agitated the solution to bend ZnO–WO_3–*x*_ nanorods to generate a piezoelectric electric field in ZnO and found that as the agitation rate increased, the PEC activity of the Z‐scheme heterojunction improved. Additionally, the bending of the ZnO–WO_3–*x*_ nanorods was controlled by changing the direction of the solution flow. When the direction of the solution flow was parallel to the nanorods, the performance of the nanorods was not significantly changed due to the insignificant deformation of the nanorods. When the solution flow direction was perpendicular to the nanorods, the nanorods were obviously bent, which significantly improved the performance. The physical mechanisms of the vertical flow of the solution are shown in Figure 6e–f. When the solution flowed perpendicularly to the ZnO nanorods, the nanorods bent, and a built‐in field was generated inside the nanorods. The built‐in field helped the photogenerated holes arrive at the surface of the ZnO to cause oxidation reactions. When the solution flowed vertically, the bending of the ZnO–WO_3–*x*_ nanorods not only generated a built‐in electric field in ZnO but also changed the band structure at the interface between ZnO and WO_3–*x*_. As positively polarized electrons accumulated on the surface of ZnO, the Fermi level of ZnO was close to that of CB, which further strengthened the transmission mechanism of the Z‐scheme heterojunction and improved the behavior of the PEC. As ultrasound is generally used as the external pressure, the direction of the piezoelectric field cannot be well controlled. Therefore, simple linear sweep voltammetry cannot describe the piezoelectric effect very well. Xu and co‐workers^[^ ^139^ ^]^ utilized a Au_4_/BaTiO_3_ heterostructure by measuring the degradation of organic dyes under auxiliary piezoelectric potential to prove that ultrasonic excitation inducing a piezoelectric field enhances the photocatalytic effect. The amount of sonophotocatalytic degradation of Au_4_/BaTiO_3_ in 75 min was greater than that of photocatalytic degradation and sonocatalytic degradation.

Therefore, piezoelectric materials are used to construct heterojunctions, and ultrasound is used to impart external force to semiconductors, causing the occurrence of polarization fields because of the redistribution of polarization charges. Reasonable utilization of a polarized electric field can effectively improve the carrier transport of semiconductor heterojunctions.

#### 4.3.3 Pyroelectric Effect

When the spontaneous polarization in a material changes with temperature, the surface of the material generates positive and negative polarization charges gathering in different areas along a certain direction.^[^ ^140^ ^]^ This characteristic is defined as the pyroelectric effect, and a material with this pyroelectric effect is a pyroelectric material. The discharge effect can be divided into the first‐level pyroelectric effect, the second‐level pyroelectric effect, and the third‐level pyroelectric effect.^[^ ^141^ ^]^ The so‐called first‐level pyroelectric effect means that the material maintains a constant strain, that is, a pyroelectric effect is derived from the polarization caused by temperature changes. The second‐level pyroelectric effect is a pyroelectric effect that occurs when the material is subjected to a constant stress. This is the polarization caused by the deformation of the material. The third‐level pyroelectric effect is the polarization induced by the gradient temperature of the material and the shear stress.

**Figure 7:**
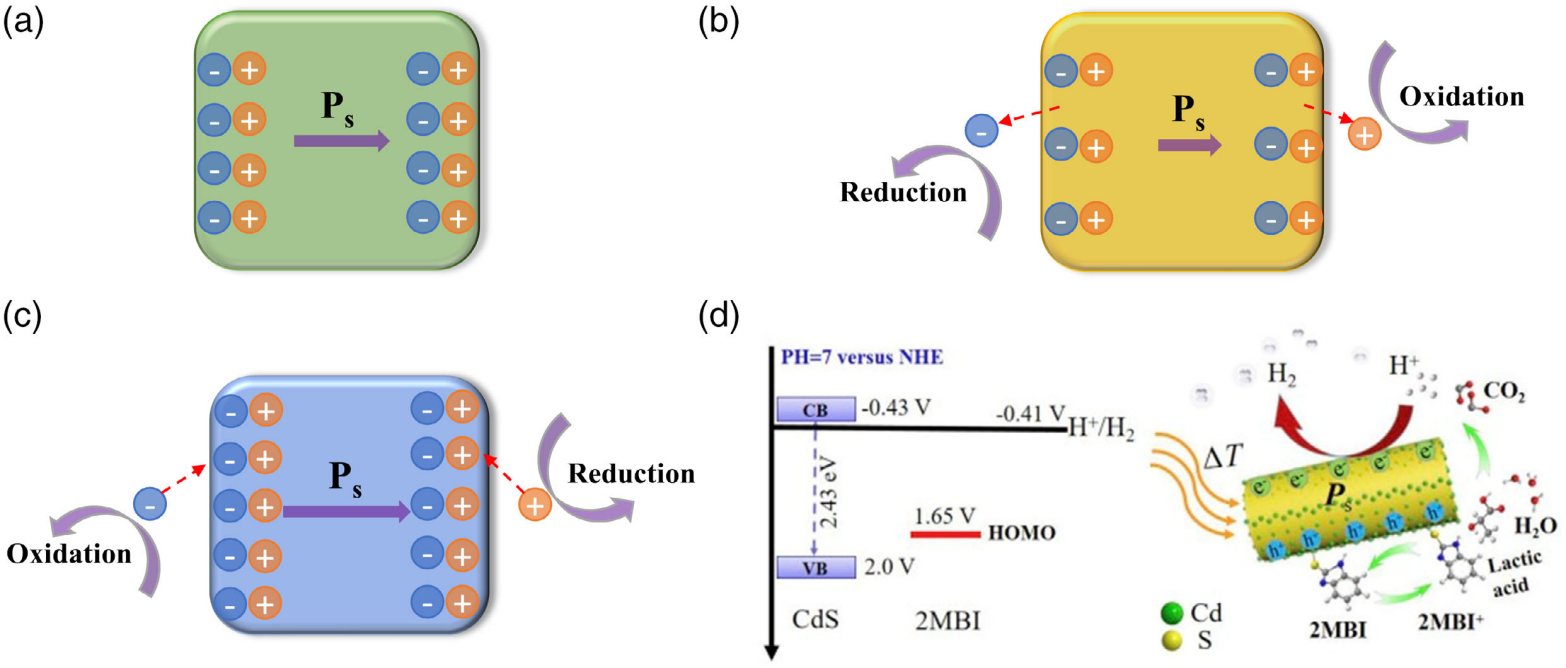
Piezoelectric semiconductors a) without temperature change, b) under warming up, and c) cooling down. d) Schematic description of the energy band and pyroelectric catalysis hydrogen evolution. Reproduced with permission.^[^ ^144^ ^]^ Copyright 2020, Elsevier.

When a pyroelectric material is heated, its electric dipoles vibrate violently, and the spontaneous polarization is weakened so that the free charge generated on the surface is redistributed and electrons are released outward. In contrast, when a pyroelectric material is cooled, the electric dipole vibrates slightly along the polarization axis to increase the spontaneous polarization and absorb electrons from the outside.^[^ ^142^ ^]^ The specific diagram is shown in **Figure** **7**a–c. In the process of the PEC photolysis of water, a photoanode can use the pyroelectric effect to make the thermally generated charges participate in the redox reactions of water. The voltage or current generated by the pyroelectric effect has the following relationship^[^ ^143^ ^]^

(5) $${I\;=\;P}_{c}\cdot \;A\;\cdot \frac{dT}{\text{dt}}$$

(6) $$V\;=\;\frac{P_{c}\cdot \;h\;\cdot \Delta T}{\varepsilon_{0}\varepsilon_{33}}$$

where *A* is the surface area, d*T*/dt refers to the temperature change rate, *P*_c_ denotes the pyroelectric coefficient, *h* is the sample thickness, Δ*T* represents the temperature change, *ε*_0_ is defined as the vacuum dielectric constant, and *ε*_33_ represents the relative dielectric constant of the material. The pyroelectric current is in direct proportion to the temperature change rate and surface area, and the pyroelectric voltage is positively related to the temperature change and the thickness of the sample. Therefore, as long as the pyroelectric material is thick enough and the temperature changing rate is fast, the polarization potential that meets the requirements of the photolysis water can be obtained, the reaction rate of the photolysis of water can be accelerated, and the performance can be improved. As shown in Figure 7d, Zhang et al.^[^ ^144^ ^]^ constructed the CdS‐2‐mercaptobenzimidazole (2MBI) heterojunction to improve its hydrogen production capacity under cooling and heating cycles, which was attributable to the pyroelectric effect of CdS, enhancing the built‐in field and broadening the width of the depletion layer to promote the transport and separation of carriers. Dai et al.^[^ ^145^ ^]^ depended on the photothermal effect of carbon nanotubes to provide temperature oscillation to the poly(vinylidene fluoride‐*co*‐hexafluropropylene) microfiber to generate a polarized electric field on the surface of the microfiber. TPC, EIS, and TRPL photoluminescence proved that the existence of a pyroelectric field greatly inhibited carrier recombination, which was the key to increasing the hydrogen production rate. For the first time, Li and co‐workers^[^ ^146^ ^]^ prepared FeS_2_/Bi_2_S_3_ for photocatalytic hydrogen production by combining the photothermal effect of FeS_2_/Bi_2_S_3_ and circulating condensed water to provide temperature vibration to the pyroelectric semiconductor Bi_2_S_3_. In this way, the direction of the Bi_2_S_3_ pyroelectric field was controlled, and FeS_2_/Bi_2_S_3_ stimulated the photogenerated electrons of FeS_2_ to migrate to the surface of Bi_2_S_3_ under the influence of the positive polarization field, which boosted the decomposition of water to produce hydrogen.

The pyroelectric catalytic process requires the following indispensable conditions: one is a pyroelectric catalyst with sufficient thickness, another is a temperature gradient, and the third is that the working temperature is below the Curie temperature.^[^ ^147^ ^]^ Only when there are temperature fluctuations in the pyroelectric body and they are below the Curie temperature, it is possible for the pyroelectric free charges with catalytic activity to be generated. At the same time, the thickness of the catalyst is positively correlated with the pyroelectric potential difference, which also affects the oxidation–reduction reaction. Therefore, while constructing heterojunctions, materials with pyroelectric effects are selected, or some modification methods (such as element substitution, construction of ultrathin structures, etc.) are used to change the symmetry of the materials to have pyroelectric properties, which contribute to the catalytic ability of the heterostructure.

#### 4.3.4 Ferroelectric Effect

**Figure 8:**
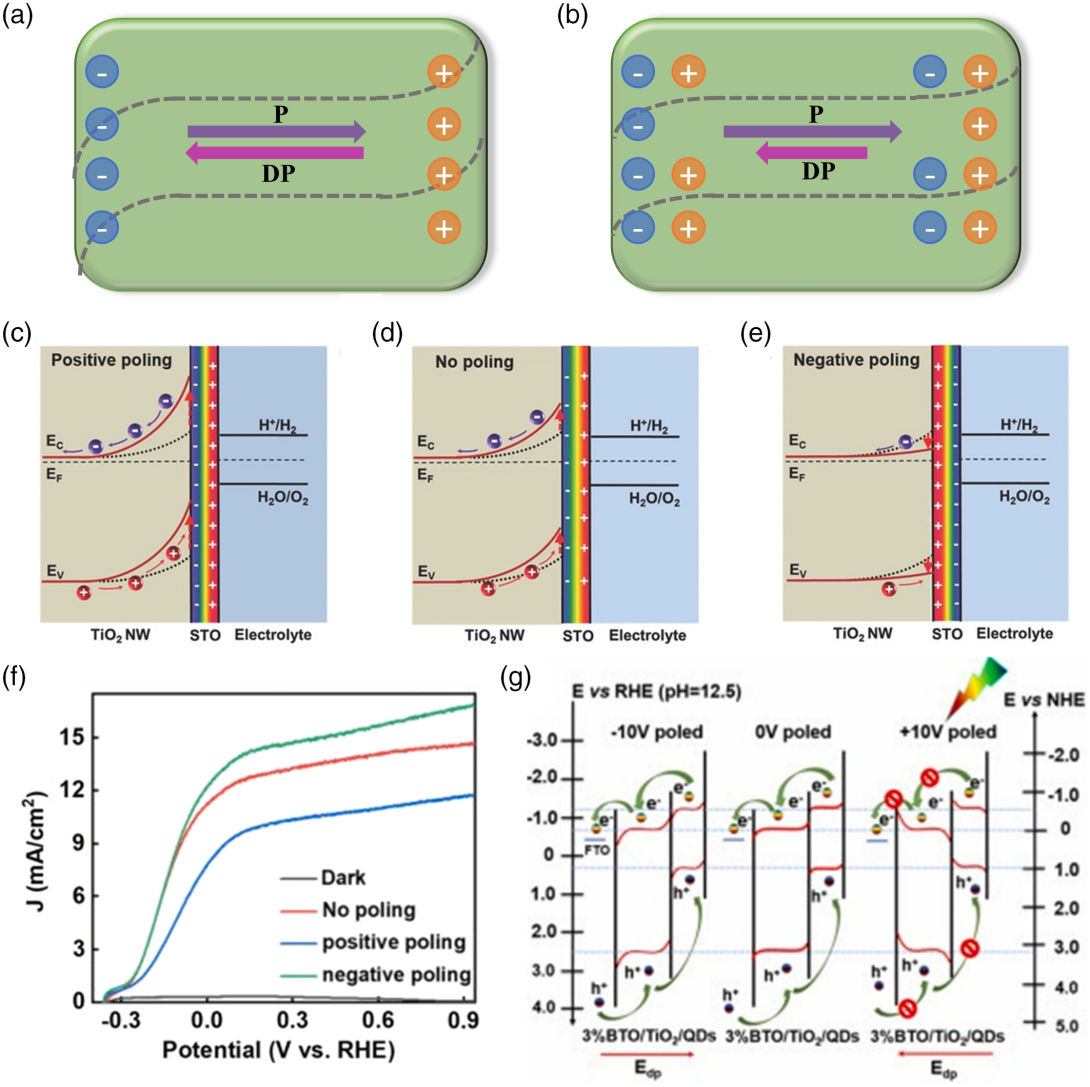
a) Schematic diagram of the depolarization field generated by the polarization charges of the ferroelectric material without compensation. b) Schematic of the depolarization field generated by the polarization charges of the ferroelectric material with partial compensation. Descriptions of band bending with c) positive poling, d) no poling, and e) negative poling. Reproduced with permission.^[^ ^152^ ^]^ Copyright 2017, Wiley‐VCH. f) Schematic depictions of *J–V* curves and g) electron transfer diagrams of 3 wt% BTO/TiO_2_/QDs with positive poling, no poling, and negative poling. Reproduced with permission.^[^ ^153^ ^]^ Copyright 2021, Elsevier.

Pyroelectric crystals can be classified into two categories. One is a pyroelectric crystal whose energy is a minimum value, and the interaction between parallel dipoles is very strong in most cases, and it is difficult to change its direction. Another type is that when the parallel dipoles are not so strongly bound to each other, the polarization direction is easily reversed by the electric field, which is called ferroelectrics.^[^ ^148^ ^]^ When the action of the polarization field belongs only to the ferroelectric material itself, once the electrode and the substrate at the interface cannot provide enough free charge to compensate for the polarization charge, a depolarization electric field opposite to the polarization field will be generated, which can also be understood as an internal electric field.^[^ ^149^ ^]^ The appearance of this polarization field will affect the band bending degree. The accumulation of positive polarity charges impels downward bending of the energy band, and the collection of negative polarity charges induces upward bending of the energy band, as shown in **Figure** **8**a,b.^[^ ^150^ ^]^ Therefore, if an applied external electric field is consistent with the polarization field direction of the ferroelectric material, the built‐in field intensity will be enhanced, and if an applied external electric field is reversed with its own polarization field direction, the built‐in electric field intensity will be reduced.^[^ ^151^ ^]^ However, the compounds of semiconductor materials and ferroelectric materials are influenced not only by the built‐in electric field but also by band bending at the interface. At the same time, when positive charges appear at the interface, they will cause the energy band to bend downward, resulting in an increase in the depletion layer in the ferroelectric. When negative charges appear at the interface, the energy band bends upward, resulting in a decrease in the depletion layer in the ferroelectric. Wu and co‐workers^[^ ^152^ ^]^ compounded SrTiO_3_ on the surface of TiO_2_ due to lattice distortion to induce the iron polarization of SrTiO_3_, which applied a positive polarization field to improve performance and applied a negative polarization field to decrease performance. When positive poling was applied, the built‐in electric field was consistent with the transfer direction of charge, and the driving force was enhanced. When negative poling was applied, as the SrTiO_3_ is only 10 nm thick, the band bending direction was not changed, but the built‐in electric field intensity was weakened. In the case of positive and negative polarization, the distribution of polarization charges, the built‐in electric field and energy band bending, and the corresponding performance characteristics are presented in detail in Figure 8c–e. However, Zhang et al.^[^ ^153^ ^]^ prepared a BaTiO_3_/TiO_2_/graded quantum dots (QDs) photoelectrode, and the photocurrent density values on applying positive and negative electric fields were reduced and improved, respectively, as shown in Figure 8f–g. Because the BaTiO_3_ nanoparticles were ≈208 nm, when the BaTiO_3_/TiO_2_/graded QDs photoelectrodes were negatively polarized, the band bending direction and the increase in the depletion layer in the BaTiO_3_ body were beneficial to the separation and transport of the photogenerated electrons. However, when the BaTiO_3_/TiO_2_/graded QDs photoelectrodes were positively polarized, the band bending direction changed greatly, which suppressed the performance.

Because the piezoelectric effect, pyroelectric effect, and ferroelectric effect all promote the enhancement of the built‐in electric field in the heterojunctions and increase the width of the depletion layer, the application of a variety of effects in the heterojunction can achieve shocking results. For example, Zhao et al.^[^ ^154^ ^]^ applied ultrasonic vibration and temperature changes to the Ag_2_O/BaTiO_3_ heterojunction, and the pollution degradation time was significantly increased, proving that the piezoelectric and pyroelectric effects played an important role in the heterojunction. Liu et al.^[^ ^155^ ^]^ added a BaTiO_3_ layer between Ag_2_O and TiO_2_, which applied forward polarization to generate a piezoelectric field and enhanced the photogenerated carrier lifetime. **Table** **3** summarizes the catalytic performance of heterojunctions with special physical effects.

**Table 3:** Summary of PEC performance for heterojunctions aided by different physical effects

Sample	Physical effects	Light condition	*J* (1.23 V_RHE_)	Testing condition	Catalytic activity	Ref.
Cu_2_S/Fe_2_O_3_‐anneal	Photothermal	UV–vis–NIR	1.19 mA cm^−2^	1 m KOH	/	[129]
NiOOH/FeOOH/Co_3_O_4_/BiVO_4_	Photothermal	AM1.5G + NIR	6.34 mA cm^−2^	0.5 m KPi	93% of Faraday efficiency for O_2_ evolution	[131]
Ni_2_S_3_/FeNi Foam	Photothermal	IR	492.2 mA cm^−2^ (1.55 V_RHE_)	1 m KOH	/	[132]
Ni‐Co_3_O_4_	Photothermal	808 nm laser of 5 W cm^−2^	441.8 mA cm^−2^ (1.6 V_RHE_)	1 m KOH	/	[133]
Pt/ZnO/Co‐Pi	Piezoelectric	AM1.5G 90 kHz ultrasonic vibrations	0.80 mA cm^−2^	0.1 m Na_2_SO_4_ 0.1 m KPi	/	[137]
ZnO‐WO_3–*x*_	Piezoelectric	AM1.5G 1000 rpm magnetic stir	3.38 mA cm^−2^	1 m Na_2_SO_4_	/	[138]
Au_4_/BaTiO_3_	Piezoelectric	AM1.5G and ultrasonic	/	/	MO degradation rate of 97% in75 min	[139]
CdS‐2MBI	Pyroelectric	0.05 mW m^−2^ light 25–55 °C (12 °C min^−1^)	/	/	H_2_ yield of 4.3 μmol g^−1^ perthermal cycle	[144]
PVDF‐HFP/CNT/CdS	Pyroelectric	280 W Xenon lamp	/	/	H_2_ evolution rate of451 μmol g^−1^ h^−1^	[145]
FeS_2_/Bi_2_S_3_	Pyroelectric	AM1.5G 22.7–33.9 °C (2.24 °C min^−1^)	/	/	H_2_ evolution rate of16.8 mmol g^−1^ h^−1^	[146]
TiO_2_–SrTiO_3_	Ferroelectric	AM1.5G ± 10 V poling voltage	1.43 mA cm^−2^	1 m NaOH	/	[152]
BaTiO_3_/TiO_2_/graded QDs	Ferroelectric	AM1.5 G ± 10 V DC voltages	15.3 mA cm^−2^ (0.5 V_RHE_)	0.25 m Na_2_S 0.35 m Na_2_SO_3_	H_2_ evolution rate of96.5 mL cm^−2^ d^−1^	[153]
Ag_2_O/BaTiO_3_	Piezoelectric Pyroelectric	AM1.5G Ultrasonic vibrations 20–50 °C (1 °C min^−1^)	3.48 mA cm^−2^ (0.5 V_SCE_)	0.1 m Na_2_SO_4_	MO degradation rate of nearly100% in 60 min	[154]
TiO_2_/ BaTiO_3_/Ag_2_O	Piezoelectric Ferroelectric	150 W Xenon lamp A bias of 0.3 V	1.8 mA cm^−2^ (0.8 V_Ag/Cl_)	1 m NaOH	/	[155]

### 4.4 Intermediate Layer

Generally, heterojunctions have two fundamental problems: lattice mismatch and energy band mismatch, which are extremely detrimental to charge transport in the bulk.^[^ ^156^ ^]^ Therefore, adding an intermediate layer between the two heterojunctions can relieve these problems. Generally, the heterojunction photoelectrode has an interface between FTO and the semiconductor, a contact interface between the heterojunction interfaces, and an interface between the photoanode and the electrolyte. This section mainly focuses on adding intermediate layers at these three interfaces to achieve PEC performance improvements.

**Figure 9:**
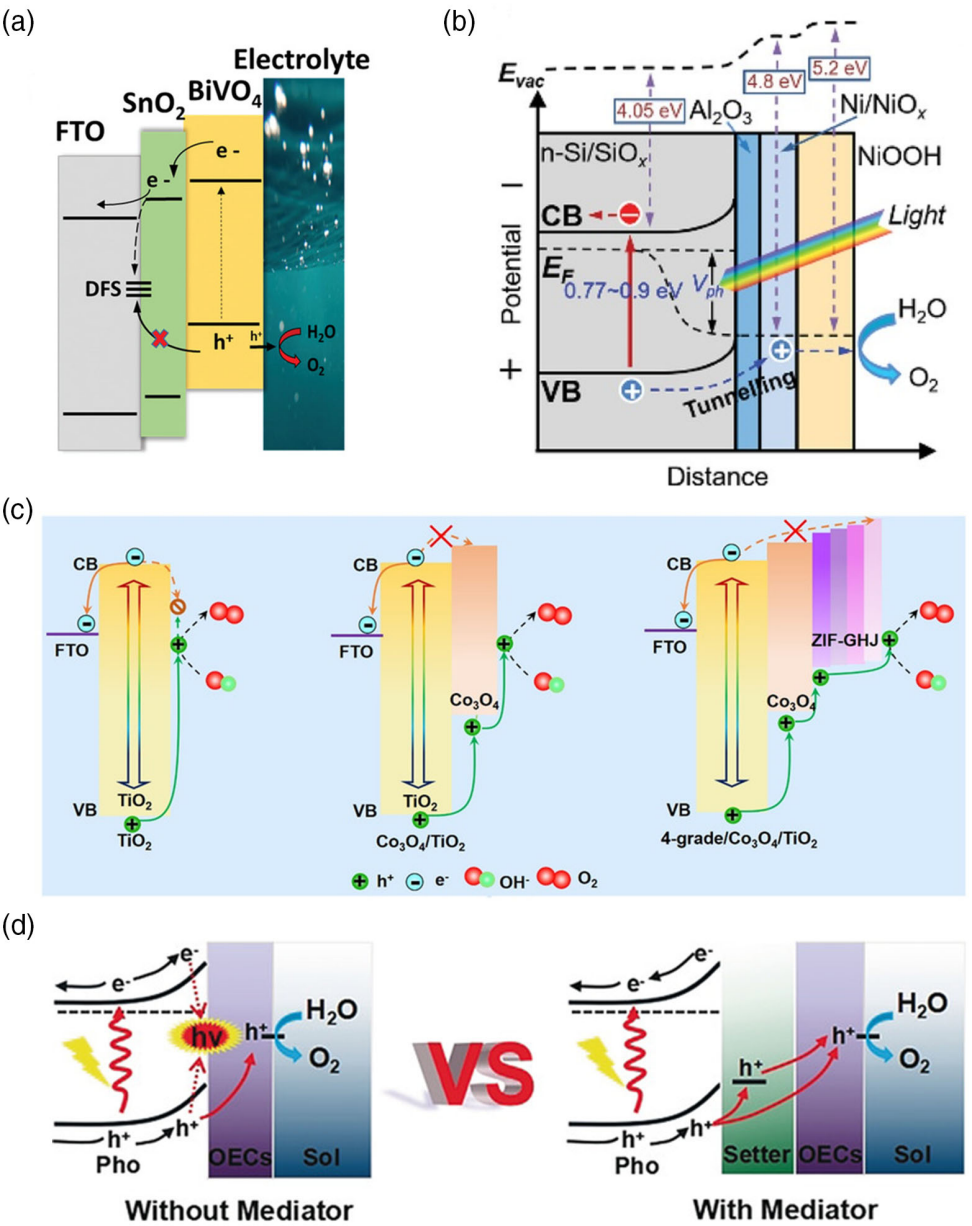
a) Schematic depiction of the band energy positions after the introduction of SnO_2_. Reproduced with permission.^[^ ^157^ ^]^ Copyright 2011, American Chemical Society. b) Band structure of the n‐Si/SiO_*x*_/Al_2_O_3_/Ni/NiO_*x*_/NiOOH photoanode. Reproduced with permission.^[^ ^161^ ^]^ Copyright 2019, Wiley‐VCH. c) Schematic diagrams showing the introduction of ZIF‐GHJ as a photogenerated hole extraction layer at the photoanode/electrolyte interface. Reproduced with permission.^[^ ^166^ ^]^ Copyright 2021, Wiley‐VCH. d) An energetics schematic showing the photogenerated carrier transport process of composite photoanodes without a mediator and with a mediator. Reproduced with permission.^[^ ^167^ ^]^ Copyright 2019, Wiley‐VCH.

When complex defect states are generated at the interface between the photoanode and the FTO, the defect states become recombination centers of photogenerated pairs of electrons and holes, which seriously affect the photocatalytic performance of photoanodes. At the FTO/BiVO_4_ interface, the defect states trapped a large number of electrons because the defect states acted as deep‐level defects in the FTO and the accumulated electrons flattened the energy band, which promoted the back contact of the photogenerated holes and resulted in serious recombination. As shown in **Figure** **9**a, the introduction of SnO_2_ into FTO/BiVO_4_ by Liang and co‐workers^[^ ^157^ ^]^ passivated the defects at the interface, inhibited the back transport of photogenerated holes, and achieved effective separation between photogenerated electrons and holes, thereby improving its PEC performance. In addition, the lattice mismatch between the FTO and the semiconductor makes the interface contact between the semiconductor and the FTO poor. A seed layer can be introduced for the transition, which can not only provide a bridge for carrier transport but also achieve directional growth of crystals. Chen et al.^[^ ^158^ ^]^ controlled the band arrangement between the anatase TiO_2_ seed layer and rutile TiO_2_ nanorods, bringing about enhanced photogenerated carrier separation performance and better electron storage efficiency at the TiO_2_/FTO interface, as well as an excellent electron transfer rate from defect states in the CdS QDs to the CB of the TiO_2_ in TiO_2_/CdS heterostructure.

As the CB and VB band positions of the two semiconductors are quite different from each other, which is unfavorable to the transportation of photogenerated electrons and holes, an intermediate layer is added at the interface of the heterojunction to achieve a graded transition to promote the bulk phase separation of the carriers. Cao et al.^[^ ^159^ ^]^ showed that the stepped band structure between ZnO, CdS, and ZnFe_2_O_4_ improved the separation efficiency of photogenerated charges. Pan et al.^[^ ^76^ ^]^ achieved a double type‐II heterojunction by adding a TiO_2_ layer between SnO_2_ and BiVO_4_ to promote the separation of charges and carriers in the bulk. In addition to the impact of band mismatching, there may also be interface contact problems. Therefore, optimizing interface contact and reducing recombination can also be achieved through the use of an intermediate layer.^[^ ^160^ ^]^ In Figure 9b, Luo and co‐workers^[^ ^161^ ^]^ deposited Al_2_O_3_ on the interface between Si/SiO_*x*_ and Ni/NiO_*x*_ by atomic layer deposition, which passivated the interface defects and effectively reduced the pinning effect of the Fermi level.

In view of the photoanode/electrolyte interface, the carriers may accumulate on the surface to cause serious interface recombination and affect performance due to electron back transfer and sluggish transfer dynamics.^[^ ^162^, ^163^, ^164^, ^165^ ^]^ Therefore, an intermediate layer is added to promote carrier transport for the water splitting redox reaction. Tang et al.^[^ ^166^ ^]^ found that TiO_2_ forced electrons to be transported to the electrolyte interface, causing serious carrier loss. By constructing a Co_3_O_4_/TiO_2_ heterojunction, the back‐transport phenomenon of the TiO_2_ electrons was suppressed, and the matching type‐II heterojunction band structure promoted the transport of the holes to the electrolyte. As shown in Figure 9c, considering that ZIF‐Co_*x*_Zn_1–*x*_ had a more negative VB position than Co_3_O_4_, the driving force for hole extraction in the ZIF‐Co_*x*_Zn_1–*x*_/Co_3_O_4_/TiO_2_ multigraded heterojunction was enhanced, which was conducive to the rapid transfer of holes to the photoanode/electrolysis interface and further inhibited the recombination of the carriers. Ning et al.^[^ ^167^ ^]^ found that there was a large potential difference between the VB of reduced BiVO_4_ and the potential of splitting water to produce oxygen. Therefore, in **Figure** 9d, 5,10,15,20‐tetrakis(4‐carboxyphenyl)porphyrin‐Co(CoPy) acted as a transport channel, similar in action to that of a volleyball setter, to advance the transfer rate of photogenerated holes reaching the surface of the photoanode, and IMPS certified that BiVO_4_/CoPy/FeNi(OH)_*x*_ was longer than BiVO_4_/FeNi(OH)_*x*_ in accordance with *τ*_d_ = (2*πf*_max_)^−1^, in which CoPy facilitated hole transmission from BiVO_4_ to FeNi(OH)_*x*_ and avoided the accumulation of carriers at the photoanode/electrolysis interface.

Therefore, in light of the unique problems of the selected semiconductors, such as large energy band differences, interface defects, and other problems, it is easy to cause carrier losses, and the introduction of an intermediate layer can be used as a transition, thereby realizing the effective transmission and separation of carriers and improving the performance of photoanode PEC water splitting. **Table** **4** briefly summarizes some heterojunctions with different functional interlayers.

**Table 4:** Summary of PEC performance for heterojunctions with intermediate layers

Sample	Intermediate layer	*J* (1.23 V_RHE_)	IPCE	Testing condition	Ref.
FTO/SnO_2_/BiVO_4_	Seed layer	/	46% at 450 nm, 1.63 V_RHE_	0.15 m K_2_SO_4_	[157]
TiO_2_‐SC‐c/CdS	Seed layer	6.80 mA cm^−2^ (1.0 V_RHE_)	/	0.25 m Na_2_S/0.35 m Na_2_SO_3_	[158]
ZnFe_2_O_4_/CdS/ZnO	Protection layer	3.88 mA cm^−2^ (0 V_Ag/AgCl_)	/	0.5 m Na_2_S	[159]
SnO_2_/TiO_2_/BiVO_4_/FeOOH/NiOOH	Hole blocking layer	3.1 mA cm^−2^	85% at 350 nm, 1.23 V_RHE_	0.5 m Na_2_SO_4_/0.1 m Na_2_SO_3_	[76]
n‐Si/SiO_*x*_/Al_2_O_3_/Ni/NiO_*x*_/NiOOH	Passivation layer	28 mA cm^−2^	70% at 420–800 nm 1.23 V_RHE_	1 m Na_2_SO_4_/0.1 m KPi	[161]
ZIF‐Co_*x*_Zn_1–*x*_/Co_3_O_4_/TiO_2_	Hole extraction layer	2.91 mA cm^−2^	/	1 m NaOH	[166]
R‐BiVO_4_/CoPy/FeNi(OH)_*x*_	Passivation layer Hole extraction layer	4.75 mA cm^−2^	/	0.2 m Na_2_SO_4_	[167]

Regarding the four strategies for promoting carrier transport in the above analysis, the following four points need to be clarified: first, micro–nanostructures are diverse and each material has several conventional morphologies due to its intrinsic crystallographic structure, so it is a certain challenge to synthesize new micro–nanostructures to some extent. Moreover, the nanostructure is selectively fine‐tuned according to the specific characteristics of the material. For example, the hole diffusion length of BiVO_4_ is about 70 nm, so the synthesized BiVO_4_ is often a hollow structure rather than a bulk structure. Second, the construction of type‐II and Z‐scheme heterojunctions requires the selection of semiconductors that match the energy bands as far as possible, while the element selection of gradient doping and conventional preparation methods have certain limitations. Third, semiconductors with specific structural characteristics have special physical effects. Fourth, the selection of the intermediate layer needs to be determined according to the specific situation, and the preparation method should avoid introducing more defect states.

## 5 Conclusion

In conclusion, we summarized some strategies that can be used to improve carrier transport in heterojunctions. First, some neatly arranged nanotopographies are chosen as promising candidates for photoelectrodes because they can increase light absorption and improve the carrier transport channel. Second, by constructing band‐matched heterojunctions, such as type‐II and Z‐scheme heterojunctions, bulk carrier transport is promoted. In addition, effective bulk phase separation of heterojunctions can also be achieved with the help of gradient doping. Then, by utilizing the photothermal effect, piezoelectric effect, and other effects to further accelerate the carrier bulk transfer rate, improved photoelectric catalytic performance was achieved. Finally, we introduced the intermediate layer, which plays the roles of electron transport layer, hole blocking layer, energy‐level transition, etc. to enhance the photogenerated carrier separation and transport capability in the heterojunctions. To monitor carrier transmission, characterization methods, such as TRPL, TAS, TPV, TPC, IMPS, and IMVS, are provided in this review for characterizing the behavior of carriers in heterojunctions.

It is not common for the construction of photoelectrodes to use only a single semiconductor. In contrast, the combination of multiple semiconductors has become the main trend of photoelectrode construction. Therefore, the successful construction of an effective heterojunction has become an urgent problem to be solved. The construction of efficient heterojunctions must take into account the factors of lattice constants, thermal expansion coefficients, and band positions, which depend on the material itself. Lattice mismatch and different thermal expansion coefficients tend to introduce a large number of interface states during the construction of heterojunctions, resulting in nonradiative recombination. If the epitaxial layer below the critical thickness is grown by atomic layer epitaxy, the lattice mismatch can be compensated by elastic strain. Therefore, first, it is necessary for the construction of heterostructures to correctly select semiconductor materials and growth techniques. Second, in situ growth and one‐step synthesis can serve as the preferred options for the construction of heterojunctions. Finally, type‐II and Z‐scheme heterostructures are the most advantageous heterojunction types at present. It is vital to select semiconductors with suitable energy bands for compound heterostructures. However, semiconductors that are not fully adapted can be modified and regulated by introducing heteroatoms or defects so as to meet the requirements of energy band matching. This review has launched a systematic and detailed discussion and summary on the carrier transport problem of heterojunctions.

Due to the current serious problems of energy shortages and environmental pollution, PEC production capacity and pollutant degradation show great advantages over many other solutions. The key contents of PEC water splitting are light absorption, the competition between the separation and recombination of photogenerated electron–hole pairs, and the reaction kinetics of the OER. With the discovery of nanomaterials with 2D layered structures, vertical and in‐plane heterostructures received great attention from researchers. The construction of vertical heterojunctions mainly uses mechanical transfer technology to stack layered 2D materials in the vertical direction, and the layers are combined by van der Waals forces, which has nearly perfect interfaces. Therefore, van der Waals heterojunctions provide an ideal platform for the exploration and understanding of heterojunctions and may be another possible route for photoelectrocatalysis. An in‐plane heterojunction refers to a composite material in which two 2D layered materials are bonded by chemical bonds in a plane. The bonding force of the chemical bond is much larger than the van der Waals force, so in‐plane heterojunctions are superior to the vertical heterojunctions in driving the separation and migration of photogenerated charges. However, the construction of planar heterojunctions is currently limited to several 2D nanomaterials due to strict lattice matching constraints and the difficulty of selecting suitable material components.^[^ ^169^ ^]^ Therefore, exploring the possibility of new 2D material heterojunction structures is crucial for promoting the development of materials field and improving solar energy conversion efficiency.

However, in order to obtain a universal modification method for the heterojunction interface problem, we need to pay more attention to the physical and chemical properties of the material, which can be exploited to improve the lattice mismatch, thermal expansion coefficient mismatch, and energy band mismatch. Of course, mining new nanomaterials and preparation methods is also an important part of current heterojunction engineering. Therefore, the exploration based on the mechanism of improving the heterojunction interface problem will become the main direction of future research, rather than simply selecting materials for trial and stacking.

## Conflict of Interest

The authors declare no conflict of interest.

## References

[1] T. R. Cook , D. K. Dogutan , S. Y. Reece , Y. Surendranath , T. S. Teets , D. G. Nocera , Chem. Rev. 2010, 110, 6474. 21062098 10.1021/cr100246c

[2] J. Qi , W. Zhang , R. Cao , Adv. Energy Mater. 2018, 8, 1701620.

[3] R. E. Blankenship , D. M. Tiede , J. Barber , G. W. Brudvig , G. Fleming , M. Ghirardi , M. R. Gunner , W. Junge , D. M. Kramer , A. Melis , T. A. Moore , C. C. Moser , D. G. Nocera , A. J. Nozik , D. R. Ort , W. W. Parson , R. C. Prince , R. T. Sayre , Science 2011, 332, 805. 21566184 10.1126/science.1200165

[4] J. Lin , W. Wang , G. Li , Adv. Funct. Mater. 2020, 30, 2005677.

[5] P. AR , IJIRAE 2014, 2, 2349.

[6] Z. Li , M. Hu , P. Wang , J. Liu , J. Yao , C. Li , Coord. Chem. Rev. 2021, 439, 213953.

[7] Y. Qiu , Z. Pan , H. Chen , D.. Ye , L. Guo , Z. Fan , S. Yang , Sci. Bull. 2019, 64, 1348. 10.1016/j.scib.2019.07.017 36659664

[8] M. Faraji , M. Yousefi , S. Yousefzadeh , M. Zirak , N. Naseri , T. H. Jeon , W. Choi , A. Z. Moshfegh , Energy Environ. Sci. 2019, 12, 59.

[9] C. Jiang , S. J. A. Moniz , A. Wang , T. Zhang , J. Tang , Chem. Soc. Rev. 2017, 46, 4645. 28644493 10.1039/c6cs00306k

[10] H. Wu , H. L. Tan , C. Y. Toe , J. Scott , L. Wang , R. Amal , Y. H. Ng , Adv. Mater. 2020, 32, 1904717. 10.1002/adma.201904717 31814196

[11] H. Huang , B. Pradhan , J. Hofkens , M. B. J. Roeffaers , J. A. Steele , ACS Energy Lett. 2020, 5, 1107.

[12] A. Ghobadi , T. G. Ulusoy Ghobadi , F. Karadas , E. Ozbay , Adv. Opt. Mater. 2019, 7, 1900028.

[13] M. Volokh , G. Peng , J. Barrio , M. Shalom , Angew. Chem., Int. Ed. 2019, 58, 6138. 10.1002/anie.201806514 30020555

[14] R. Siavash Moakhar , S. M. Hosseini-Hosseinabad , S. Masudy-Panah , A. Seza , M. Jalali , H. Fallah-Arani , F. Dabir , S. Gholipour , Y. Abdi , M. Bagheri-Hariri , N. Riahi-Noori , Y. F. Lim , A. Hagfeldt , M. Saliba , Adv. Mater. 2021, 33, 2007285. 34117806 10.1002/adma.202007285 PMC11468279

[15] A. Fujishima , K. Honda , Nature 1972, 238, 37. 12635268 10.1038/238037a0

[16] Y. Wang , W. Tian , C. Chen , W. Xu , L. Li , Adv. Funct. Mater. 2019, 29, 1809036.

[17] Y. Yang , S. Niu , D. Han , T. Liu , G. Wang , Y. Li , Adv. Energy Mater. 2017, 7, 1700555.

[18] Q. Wang , K. Domen , Chem. Rev. 2020, 120, 919. 31393702 10.1021/acs.chemrev.9b00201

[19] Y. He , T. Hamann , D. Wang , Chem. Soc. Rev. 2019, 48, 2182. 30667004 10.1039/c8cs00868j

[20] S. M. Thalluri , L. Bai , C. Lv , Z. Huang , X. Hu , L. Liu , Adv. Sci. 2020, 7, 1902102. 10.1002/advs.201902102 PMC7080548 32195077

[21] X. Zhang , H. Guo , G. Dong , Y. Zhang , G. Lu , Y. Bi , Appl. Catal., B 2020, 277, 119217.

[22] M. Kim , B. Lee , H. Ju , J. Y. Kim , J. Kim , S. W. Lee , Adv. Mater. 2019, 31, 1903316.

[23] X. Zhou , P. Gai , P. Zhang , H. Sun , F. Lv , L. Liu , S. Wang , ACS Appl. Mater. Interfaces 2019, 11, 38993. 31556586 10.1021/acsami.9b12560

[24] H. Zhao , X. Li , M. Cai , C. Liu , Y. You , R. Wang , A. I. Channa , F. Lin , D. Huo , G. Xu , X. Tong , Z. M. Wang , Adv. Energy Mater. 2021, 11, 2101230.

[25] B. Zhang , H. Zhang , Z. Wang , X. Zhang , X. Qin , Y. Dai , Y. Liu , P. Wang , Y. Li , B. Huang , Appl. Catal., B 2017, 211, 258.

[26] M. Sun , R. T. Gao , J. He , X. Liu , T. Nakajima , X. Zhang , L. Wang , Angew. Chem., Int. Ed. 2021, 60, 17601. 10.1002/anie.202104754 34018300

[27] Z. Wu , Y. Zhao , W. Jin , B. Jia , J. Wang , T. Ma , Adv. Funct. Mater. 2021, 31, 2009070.

[28] L. Ran , S. Qiu , P. Zhai , Z. Li , J. Gao , X. Zhang , B. Zhang , C. Wang , L. Sun , J. Hou , J. Am. Chem. Soc. 2021, 143, 7402. 33961743 10.1021/jacs.1c00946

[29] Y. Pihosh , V. Nandal , T. Minegishi , M. Katayama , T. Yamada , K. Seki , M. Sugiyama , K. Domen , ACS Energy Lett. 2020, 5, 2492.

[30] Y. Wu , X. Liu , H. Zhang , J. Li , M. Zhou , L. Li , Y. Wang , Angew. Chem., Int. Ed. 2021, 60, 3487. 10.1002/anie.202012734 33128336

[31] S. Liu , R. Gao , M. Sun , Y. Wang , T. Nakajima , X. Liu , W. Zhang , L. Wang , Appl. Catal., B 2021, 292, 120063.

[32] B. Zhang , X. Huang , Y. Zhang , G. Lu , L. Chou , Y. Bi , Angew. Chem., Int. Ed. 2020, 59, 18990. 10.1002/anie.202008198 32666681

[33] F. Li , H. Yang , Q. Zhuo , D. Zhou , X. Wu , P. Zhang , Z. Yao , L. Sun , Angew. Chem., Int. Ed. 2021, 60, 1976. 10.1002/anie.202011069 PMC7894348 33051952

[34] Y. Yu , Y. Huang , Y. Yu , Y. Shi , B. Zhang , Nano Energy 2018, 43, 236.

[35] H. Zhang , D. Li , W. J. Byun , X. Wang , T. J. Shin , H. Y. Jeong , H. Han , C. Li , J. S. Lee , Nat. Commun. 2020, 11, 4622. 32934221 10.1038/s41467-020-18484-8 PMC7493915

[36] G. Liu , M. Wang , H. Wang , R. E. A. Ardhi , H. Yu , D. Zou , J. K. Lee , Nano Energy 2018, 49, 95.

[37] J. H. Kim , J. S. Lee , Adv. Mater. 2019, 31, 1806938.

[38] M. Ma , K. Zhang , P. Li , M. S. Jung , M. J. Jeong , J. H. Park , Angew. Chem., Int. Ed. 2016, 55, 11819. 10.1002/anie.201605247 27533279

[39] S. Singh , H. Chen , S. Shahrokhi , L. Wang , C. Lin , L. Hu , X. Guan , A. Tricoli , Z. Xu , T. Wu , ACS Energy Lett. 2020, 5, 1487.

[40] J. Su , T. Hisatomi , T. Minegishi , K. Domen , Angew. Chem., Int. Ed. 2020, 59, 13800. 10.1002/anie.202000688 32394584

[41] D. Lee , A. Kvit , K. Choi , Chem. Mater. 2018, 30, 4704.

[42] H. Zhu , Q. Yang , D. Liu , D. Liu , W. Zhang , Z. Chu , X. Wang , S. Yan , Z. Li , Z. Zou , J. Phys. Chem. Lett. 2020, 11, 9184. 33058679 10.1021/acs.jpclett.0c02291

[43] D. Chen , Z. Liu , S. Zhang , Appl. Catal., B 2020, 265, 118580.

[44] Y. Fu , Y. Lu , F. Ren , Z. Xing , J. Chen , P. Guo , W. Pong , C. Dong , L. Zhao , S. Shen , Solar RRL 2019, 4, 1900349.

[45] Y. Gu , A. Wu , Y. Jiao , H. Zheng , X. Wang , Y. Xie , L. Wang , C. Tian , H. Fu , Angew. Chem. Int. Ed. 2021, 60, 6673. 10.1002/anie.202016102 33331671

[46] J. Low , J. Yu , M. Jaroniec , S. Wageh , A. A. Al Ghamdi , Adv. Mater. 2017, 29, 1601694. 10.1002/adma.201601694 28220969

[47] M. Xiao , B. Luo , Z. Wang , S. Wang , L. Wang , Solar RRL 2020, 4, 1900509.

[48] Q. Xu , L. Zhang , B. Cheng , J. Fan , J. Yu , Chem 2020, 6, 1543.

[49] Z. Wang , C. Li , K. Domen , Chem. Soc. Rev. 2019, 48, 2109. 30328438 10.1039/c8cs00542g

[50] X. An , Y. Wang , J. Lin , J. Shen , Z. Zhang , X. Wang , Sci. Bull. 2017, 62, 599. 10.1016/j.scib.2017.03.025 36659299

[51] S. Yi , Z. Wang , H. Li , Z. Zafar , Z. Zhang , L. Zhang , D. Chen , Z. Liu , X. Yue , Appl. Catal., B 2021, 283, 119649.

[52] S. Lee , J. Song , Y. R. Jo , K. S. Choi , J. Lee , S. Seo , T. L. Kim , H. W. Jang , C. Jeon , B. J. Kim , B. Kim , S. Lee , ACS Appl. Mater. Interfaces 2019, 11, 44069. 31631650 10.1021/acsami.9b12916

[53] Z. Yan , H. Sun , X. Chen , H. Liu , Y. Zhao , H. Li , W. Xie , F. Cheng , J. Chen , Nat. Commun. 2018, 9, 2373. 29915288 10.1038/s41467-018-04788-3 PMC6006371

[54] L. Zhang , J. Ran , S. Z. Qiao , M. Jaroniec , Chem. Soc. Rev. 2019, 48, 5184. 31432886 10.1039/c9cs00172g

[55] T. Kirchartz , J. A. Márquez , M. Stolterfoht , T. Unold , Adv. Energy Mater. 2020, 10, 1904134.

[56] J. Xu , X. Li , Z. Ju , Y. Sun , X. Jiao , J. Wu , C. Wang , W. Yan , H. Ju , J. Zhu , Y. Xie , Angew. Chem., Int. Ed. 2019, 58, 3032. 10.1002/anie.201807332 30137662

[57] J. Li , H. Chen , C. A. Triana , G. R. Patzke , Angew. Chem., Int. Ed. 2021, 60, 18380. 10.1002/anie.202101783 33761172

[58] Z. Wei , W. Wang , W. Li , X. Bai , J. Zhao , E. C. M. Tse , D. L. Phillips , Y. Zhu , Angew. Chem., Int. Ed. 2021, 60, 8236. 10.1002/anie.202016170 33491294

[59] Z. Wang , Y. Qi , C. Ding , D. Fan , G. Liu , Y. Zhao , C. Li , Chem. Sci. 2016, 7, 4391. 30155086 10.1039/c6sc00245e PMC6014074

[60] W. Guan , Y. Li , Q. Zhong , H. Liu , J. Chen , H. Hu , K. Lv , J. Gong , Y. Xu , Z. Kang , M. Cao , Q. Zhang , Nano Lett. 2021, 21, 597. 33258607 10.1021/acs.nanolett.0c04073

[61] T. Zhou , J. Wang , S. Chen , J. Bai , J. Li , Y. Zhang , L. Li , L. Xia , M. Rahim , Q. Xu , B. Zhou , Appl. Catal., B 2020, 267, 118599.

[62] Z. Liu , K. Deng , J. Hu , L. Li , Angew. Chem., Int. Ed. 2019, 58, 11497. 10.1002/anie.201904945 31152477

[63] S. Corby , L. Francas , S. Selim , M. Sachs , C. Blackman , A. Kafizas , J. R. Durrant , J. Am. Chem. Soc. 2018, 140, 16168. 30383367 10.1021/jacs.8b08852

[64] Y. Liu , M. Xia , L. Yao , M. Mensi , D. Ren , M. Grätzel , K. Sivula , N. Guijarro , Adv. Funct. Mater. 2021, 31, 2010081.

[65] B. Li , W. Peng , J. Zhang , J. C. Lian , T. Huang , N. Cheng , Z. Luo , W. Q. Huang , W. Hu , A. Pan , L. Jiang , G. F. Huang , Adv. Funct. Mater. 2021, 31, 2100816.

[66] D. Cardenas-Morcoso , A. Bou , S. Ravishankar , M. García-Tecedor , S. Gimenez , J. Bisquert , ACS Energy Lett. 2019, 5, 187.

[67] X. Huai , L. Girardi , R. Lu , S. Gao , Y. Zhao , Y. Ling , G. A. Rizzi , G. Granozzi , Z. Zhang , Nano Energy 2019, 65, 104020.

[68] M. Antuch , P. Millet , A. Iwase , A. Kudo , Appl. Catal., B 2018, 237, 401.

[69] G. Liu , S. Ye , P. Yan , F. Xiong , P. Fu , Z. Wang , Z. Chen , J. Shi , C. Li , Energy Environ. Sci. 2016, 9, 1327.

[70] L. Meng , J. He , X. Zhou , K. Deng , W. Xu , P. Kidkhunthod , R. Long , Y. Tang , L. Li , Nat. Commun. 2021, 12, 5247. 34475386 10.1038/s41467-021-25609-0 PMC8413305

[71] P. Xu , C. L. Gray , L. Xiao , T. E. Mallouk , J. Am. Chem. Soc. 2018, 140, 11647. 30145888 10.1021/jacs.8b04878

[72] S. Cao , Y. Wu , J. Hou , B. Zhang , Z. Li , X. Nie , L. Sun , Adv. Energy Mater. 2019, 10, 1902935.

[73] R. Tang , S. Zhou , Z. Zhang , R. Zheng , J. Huang , Adv. Mater. 2021, 33, 2005389. 10.1002/adma.202005389 33733537

[74] J. W. Yang , I. J. Park , S. A. Lee , M. G. Lee , T. H. Lee , H. Park , C. Kim , J. Park , J. Moon , J. Y. Kim , H. W. Jang , Appl. Catal., B 2021, 293, 120217.

[75] W. Tian , C. Chen , L. Meng , W. Xu , F. Cao , L. Li , Adv. Energy Mater. 2020, 10, 1903951.

[76] Q. Pan , A. Li , Y. Zhang , Y. Yang , C. Cheng , Adv. Sci. 2020, 7, 1902235. 10.1002/advs.201902235 PMC7001624 32042560

[77] K. Cho , Y. Sung , Nano Energy 2017, 36, 176.

[78] B. Dong , J. Cui , Y. Qi , F. Zhang , Adv. Mater. 2021, 33, 2004697. 10.1002/adma.202004697 34085732

[79] N. N. Vu , S. Kaliaguine , T. O. Do , Adv. Funct. Mater. 2019, 29, 1901825.

[80] W. Wang , J. Dong , X. Ye , Y. Li , Y. Ma , L. Qi , Small 2016, 12, 1469. 26779803 10.1002/smll.201503553

[81] B. Dong , J. Cui , Y. Gao , Y. Qi , F. Zhang , C. Li , Adv. Mater. 2019, 31, 1808185. 10.1002/adma.201808185 30785220

[82] S. Vanka , B. Zhou , R. A. Awni , Z. Song , F. A. Chowdhury , X. Liu , H. Hajibabaei , W. Shi , Y. Xiao , I. A. Navid , A. Pandey , R. Chen , G. A. Botton , T. W. Hamann , D. Wang , Y. Yan , Z. Mi , ACS Energy Lett. 2020, 5, 3741.

[83] F. F. Abdi , L. Han , A. H. Smets , M. Zeman , B. Dam , R. van de Krol , Nat. Commun. 2013, 4, 2195. 23893238 10.1038/ncomms3195

[84] D. Zhao , Y. Wang , C. Dong , Y. Huang , J. Chen , F. Xue , S. Shen , L. Guo , Nat. Energy 2021, 6, 388.

[85] J. Low , J. Yu , M. Jaroniec , S. Wageh , A. A. Al-Ghamdi , Adv. Mater. 2017, 29, 1601694. 10.1002/adma.201601694 28220969

[86] I. Grigioni , L. Ganzer , F. V. A. Camargo , B. Bozzini , G. Cerullo , E. Selli , ACS Energy Lett. 2019, 4, 2213.

[87] C. Pei , Y. Chen , L. Wang , W. Chen , G. Huang , Appl. Surf. Sci. 2021, 535, 147682.

[88] X. Zhao , J. Feng , J. Liu , J. Lu , W. Shi , G. Yang , G. Wang , P. Feng , P. Cheng , Adv. Sci. 2018, 5, 1700590. 10.1002/advs.201700590 PMC5908348 29721410

[89] J. J. Deng , X. X. Lv , K. Q. Nie , X. L. Lv , X. H. Sun , J. Zhong , ACS Catal. 2017, 7, 4062.

[90] F. M. Pesci , M. S. Sokolikova , C. Grotta , P. C. Sherrell , F. Reale , K. Sharda , N. Ni , P. Palczynski , C. Mattevi , ACS Catal. 2017, 7, 4990.

[91] D. E. Schipper , Z. Zhao , A. P. Leitner , L. Xie , F. Qin , M. K. Alam , S. Chen , D. Wang , Z. Ren , Z. Wang , J. Bao , K. H. Whitmire , ACS Nano 2017, 11, 4051. 28333437 10.1021/acsnano.7b00704

[92] T. H. Jeon , G. h. Moon , H. Park , W. Choi , Nano Energy 2017, 39, 211.

[93] K. H. Ye , H. Li , D. Huang , S. Xiao , W. Qiu , M. Li , Y. Hu , W. Mai , H. Ji , S. Yang , Nat. Commun. 2019, 10, 3687. 31417082 10.1038/s41467-019-11586-y PMC6695449

[94] J. Zhang , H. Ma , Z. Liu , Appl. Catal., B 2017, 201, 84.

[95] H. Chai , L. Gao , P. Wang , F. Li , G. Hu , J. Jin , Appl. Catal., B 2021, 305, 121011.

[96] C. Gao , T. Wei , Y. Zhang , X. Song , Y. Huan , H. Liu , M. Zhao , J. Yu , X. Chen , Adv. Mater. 2019, 31, 1806596. 10.1002/adma.201806596 30614566

[97] L. Meng , X. Zhou , S. Wang , Y. Zhou , W. Tian , P. Kidkhunthod , S. Tunmee , Y. Tang , R. Long , Y. Xin , L. Li , Angew. Chem., Int. Ed. 2019, 58, 16668. 10.1002/anie.201910510 31507028

[98] L. Cai , J. Zhao , H. Li , J. Park , I. S. Cho , H. S. Han , X. Zheng , ACS Energy Lett. 2016, 1, 624.

[99] S. Cao , Y. Wu , J. Hou , B. Zhang , Z. Li , X. Nie , L. Sun , Adv. Energy Mater. 2019, 10, 1902935.

[100] J. Hou , S. Cao , Y. Sun , Y. Wu , F. Liang , Z. Lin , L. Sun , Adv. Energy Mater. 2018, 8, 1701114.

[101] L. Meng , M. Wang , H. Sun , W. Tian , C. Xiao , S. Wu , F. Cao , L. Li , Adv. Mater. 2020, 32, 2002893. 10.1002/adma.202002893 32567132

[102] A. J. Bard , J. Photochem. 1979, 10, 59.

[103] H. Tada , T. Mitsui , T. Kiyonaga , T. Akita , K. Tanaka , Nat. Mater. 2006, 5, 782. 16964238 10.1038/nmat1734

[104] J. Yu , S. Wang , J. Low , W. Xiao , Phys. Chem. Chem. Phys. 2013, 15, 16883. 23999576 10.1039/c3cp53131g

[105] X. Chen , J. Wang , Y. Chai , Z. Zhang , Y. Zhu , Adv. Mater. 2021, 33, 2007479. 10.1002/adma.202007479 33448048

[106] Y. Fu , C. Dong , W. Zhou , Y. Lu , Y. Huang , Y. Liu , P. Guo , L. Zhao , W. Chou , S. Shen , Appl. Catal., B 2020, 260, 118206.

[107] S. Hou , X. Dai , Y. Li , M. Huang , T. Li , Z. Wei , Y. He , G. Xiao , F. Xiao , J. Mater. Chem. A 2019, 7, 22487.

[108] F. Liu , R. Shi , Z. Wang , Y. Weng , C. M. Che , Y. Chen , Angew. Chem., Int. Ed. 2019, 58, 11791. 10.1002/anie.201906416 31241810

[109] W. Xu , W. Tian , L. Meng , F. Cao , L. Li , Adv. Energy Mater. 2021, 11, 2003500.

[110] L. Wang , X. Zheng , L. Chen , Y. Xiong , H. Xu , Angew. Chem. Int. Ed. 2018, 57, 3454. 10.1002/anie.201710557 29377491

[111] J. Low , B. Dai , T. Tong , C. Jiang , J. Yu , Adv. Mater. 2019, 31, 1802981. 10.1002/adma.201802981 30345599

[112] X. Wang , X. Wang , J. Huang , S. Li , A. Meng , Z. Li , Nat. Commun. 2021, 12, 4112. 34226543 10.1038/s41467-021-24511-z PMC8257585

[113] S. Wei , C. Wang , X. Long , T. Wang , P. Wang , M. Zhang , S. Li , J. Ma , J. Jin , L. Wu , Nanoscale 2020, 12, 15193. 32638787 10.1039/d0nr04473c

[114] L. Meng , D. Rao , W. Tian , F. Cao , X. Yan , L. Li , Angew. Chem., Int. Ed. 2018, 57, 16882. 10.1002/anie.201811632 30371007

[115] W. Qiu , S. Xiao , J. Ke , Z. Wang , S. Tang , K. Zhang , W. Qian , Y. Huang , D. Huang , Y. Tong , S. Yang , Angew. Chem., Int. Ed. 2019, 58, 19087. 10.1002/anie.201912475 31617959

[116] A. G. Hufnagel , H. Hajiyani , S. Zhang , T. Li , O. Kasian , B. Gault , B. Breitbach , T. Bein , D. Fattakhova-Rohlfing , C. Scheu , R. Pentcheva , Adv. Funct. Mater. 2018, 28, 1804472.

[117] L. Meng , S. Wang , F. Cao , W. Tian , R. Long , L. Li , Angew. Chem., Int. Ed. 2019, 58, 6761. 10.1002/anie.201902411 30907040

[118] R. Shi , H. F. Ye , F. Liang , Z. Wang , K. Li , Y. Weng , Z. Lin , W. F. Fu , C. M. Che , Y. Chen , Adv. Mater. 2018, 30, 1705941. 10.1002/adma.201705941 29280205

[119] J. Zheng , Y. Lyu , C. Xie , R. Wang , L. Tao , H. Wu , H. Zhou , S. Jiang , S. Wang , Adv. Mater. 2018, 30, 1801773. 10.1002/adma.201801773 29920801

[120] K. Zhang , G. Zhang , J. Qu , H. Liu , Small 2018, 14, 1802760. 10.1002/smll.201802760 30350550

[121] Y. Xiao , C. Feng , J. Fu , F. Wang , C. Li , V. F. Kunzelmann , C. Jiang , M. Nakabayashi , N. Shibata , I. D. Sharp , K. Domen , Y. Li , Nat. Catal. 2020, 3, 932.

[122] Y. Yu , Y. Huang , Y. Yu , Y. Shi , B. Zhang , Nano Energy 2018, 43, 236.

[123] Z. Luo , C. Li , S. Liu , T. Wang , J. Gong , Chem. Sci. 2017, 8, 91. 28451152 10.1039/c6sc03707k PMC5304616

[124] Z. Zhang , I. Karimata , H. Nagashima , S. Muto , K. Ohara , K. Sugimoto , T. Tachikawa , Nat. Commun. 2019, 10, 4832. 31645549 10.1038/s41467-019-12581-z PMC6811569

[125] Y. Liu , C. Xiao , Z. Li , Y. Xie , Adv. Energy Mater. 2016, 6, 1600436.

[126] Q. He , Y. Wan , H. Jiang , Z. Pan , C. Wu , M. Wang , X. Wu , B. Ye , P. M. Ajayan , L. Song , ACS Energy Lett. 2018, 3, 1373.

[127] J. Zheng , Y. Lyu , R. Wang , C. Xie , H. Zhou , S. P. Jiang , S. Wang , Nat. Commun. 2018, 9, 3572. 30177720 10.1038/s41467-018-05580-z PMC6120862

[128] S. Zhang , D. Chen , Z. Liu , M. Ruan , Z. Guo , Appl. Catal., B 2020, 284, 119686.

[129] Y. Zhang , Y. Huang , S. S. Zhu , Y. Y. Liu , X. Zhang , J. J. Wang , A. Braun , Small 2021, 17, 2100320. 10.1002/smll.202100320 34151514

[130] X. Hu , J. Huang , F. Zhao , P. Yi , B. He , Y. Wang , T. Chen , Y. Chen , Z. Li , X. Liu , J. Mater. Chem. A 2020, 8, 14915.

[131] B. He , S. Jia , M. Zhao , Y. Wang , T. Chen , S. Zhao , Z. Li , Z. Lin , Y. Zhao , X. Liu , Adv. Mater. 2021, 33, 2004406. 10.1002/adma.202004406 33734506

[132] Y. Zhang , Y. Wang , H. Jiang , M. Huang , Small 2020, 16, 2002550. 10.1002/smll.202002550 32705807

[133] B. Jin , Y. Li , J. Wang , F. Meng , S. Cao , B. He , S. Jia , Y. Wang , Z. Li , X. Liu , Small 2019, 15, 1903847. 10.1002/smll.201903847 31512397

[134] J. Wu , W. Wang , Y. Tian , C. Song , H. Qiu , H. Xue , Nano Energy 2020, 77, 105122.

[135] M. B. Starr , X. Wang , Nano Energy 2015, 14, 296.

[136] Z. Liu , X. Yu , L. Li , Chinese J. Catal. 2020, 41, 534.

[137] S. Zhang , Z. Liu , M. Ruan , Z. Guo , L. E , W. Zhao , D. Zhao , X. Wu , D. Chen , Appl. Catal., B 2020, 262, 118279.

[138] Y. Chen , L. Wang , R. Gao , Y. Zhang , L. Pan , C. Huang , K. Liu , X. Chang , X. Zhang , J. Zou , Appl. Catal., B 2019, 259, 118079.

[139] S. Xu , L. Guo , Q. Sun , Z. L. Wang , Adv. Funct. Mater. 2019, 29, 1808737.

[140] L. Xiao , X. Xu , Y. Jia , G. Hu , J. Hu , B. Yuan , Y. Yu , G. Zou , Nat. Commun. 2021, 12, 318. 33436627 10.1038/s41467-020-20517-1 PMC7804252

[141] C. Wang , N. Tian , T. Ma , Y. Zhang , H. Huang , Nano Energy 2020, 78, 105371.

[142] S. Zhang , B. Zhang , D. Chen , Z. Guo , M. Ruan , Z. Liu , Nano Energy 2021, 79, 105485.

[143] H. You , Y. Jia , Z. Wu , F. Wang , H. Huang , Y. Wang , Nat. Commun. 2018, 9, 2889. 30038299 10.1038/s41467-018-05343-w PMC6056473

[144] M. Zhang , Q. Hu , K. Ma , Y. Ding , C. Li , Nano Energy 2020, 73, 104810.

[145] B. Dai , J. Fang , Y. Yu , M. Sun , H. Huang , C. Lu , J. Kou , Y. Zhao , Z. Xu , Adv. Mater. 2020, 32, 1906361. 10.1002/adma.201906361 32048360

[146] M. Li , J. Sun , G. Chen , S. Yao , B. Cong , P. Liu , Appl. Catal., B 2021, 298, 120573.

[147] X. Xu , L. Xiao , Y. Jia , Z. Wu , F. Wang , Y. Wang , N. O. Haugen , H. Huang , Energy Environ. Sci. 2018, 11, 2198.

[148] L. Yang , Y. Xiong , W. Guo , M. Zhou , K. Song , P. Xiao , G. Cao , Nano Energy 2018, 44, 63.

[149] S. Dunn , P. M. Jones , D. E. Gallardo , J. Am. Chem. Soc. 2007, 129, 8724. 17589993 10.1021/ja071451n

[150] H. Wu , H. Ling , Z. Zhang , Y. Li , L. Liang , G. Chai , Acta Phys. Sinica 2017, 66, 167702.

[151] S. Singh , A. L. Sangle , T. Wu , N. Khare , J. L. MacManus-Driscoll , ACS Appl. Mater. Interfaces 2019, 11, 45683. 31710804 10.1021/acsami.9b15317

[152] F. Wu , Y. Yu , H. Yang , L. N. German , Z. Li , J. Chen , W. Yang , L. Huang , W. Shi , L. Wang , X. Wang , Adv. Mater. 2017, 29, 1701432. 10.1002/adma.201701432 28558165

[153] M. Zhang , F. Li , D. Benetti , R. Nechache , Q. Wei , X. Qi , F. Rosei , Nano Energy 2021, 81, 105626.

[154] W. Zhao , Q. Zhang , H. Wang , J. Rong , L. E , Y. Dai , Nano Energy 2020, 73, 104783.

[155] Z. Liu , L. Wang , X. Yu , J. Zhang , R. Yang , X. Zhang , Y. Ji , M. Wu , L. Deng , L. Li , Z. L. Wang , Adv. Funct. Mater. 2019, 29, 1807279.

[156] X. Sun , D. Tiwari , D. J. Fermin , Adv. Energy Mater. 2020, 10, 2002784.

[157] Y. Liang , T. Tsubota , L. P. A. Mooij , R. van de Krol , J. Phys. Chem. C 2011, 115, 17594.

[158] Y. L. Chen , Y. H. Chen , J. W. Chen , F. Cao , L. Li , Z. M. Luo , I. C. Leu , Y. C. Pu , ACS Appl. Mater. Interfaces 2019, 11, 8126. 30726054 10.1021/acsami.8b22418

[159] S. Cao , X. Yan , Z. Kang , Q. Liang , X. Liao , Y. Zhang , Nano Energy 2016, 24, 25.

[160] P. Wang , F. Li , X. Long , T. Wang , H. Chai , H. Yang , S. Li , J. Ma , J. Jin , Nanoscale 2021, 13, 14197. 34477701 10.1039/d1nr03257g

[161] Z. Luo , B. Liu , H. Li , X. Chang , W. Zhu , T. Wang , J. Gong , Small Methods 2019, 3, 1900212.

[162] K. Zhang , B. Jin , C. Park , Y. Cho , X. Song , X. Shi , S. Zhang , W. Kim , H. Zeng , J. H. Park , Nat. Commun. 2019, 10, 2001. 31043598 10.1038/s41467-019-10034-1 PMC6494903

[163] M. Zhong , T. Hisatomi , Y. Kuang , J. Zhao , M. Liu , A. Iwase , Q. Jia , H. Nishiyama , T. Minegishi , M. Nakabayashi , N. Shibata , R. Niishiro , C. Katayama , H. Shibano , M. Katayama , A. Kudo , T. Yamada , K. Domen , J. Am. Chem. Soc. 2015, 137, 5053. 25802975 10.1021/jacs.5b00256

[164] H. Chai , P. Wang , T. Wang , L. Gao , F. Li , J. Jin , ACS Appl. Mater. Interfaces 2021, 13, 47572. 34607433 10.1021/acsami.1c12597

[165] T. Wang , X. Long , S. Wei , P. Wang , C. Wang , J. Jin , G. Hu , ACS Appl. Mater. Interfaces 2020, 12, 49705. 33104336 10.1021/acsami.0c15568

[166] R. Tang , L. Wang , M. Ying , W. Yang , A. Kheradmand , Y. Jiang , Z. Li , Y. Cui , R. Zheng , J. Huang , Small Sci. 2021, 1, 2000033. 10.1002/smsc.202000033 PMC11935825 40213357

[167] X. Ning , B. Lu , Z. Zhang , P. Du , H. Ren , D. Shan , J. Chen , Y. Gao , X. Lu , Angew. Chem., Int. Ed. 2019, 58, 16800. 10.1002/anie.201908833 31486209

[168] J. H. Kim , I. Y. Choi , J. H. Kim , J. Kim , Y. K. Kim , J. K. Kim , J. S. Lee , Small 2021, 17, 2103861.

[169] Y. Gong , J. Lin , X. Wang , G. Shi , S. Lei , Z. Lin , X. Zou , G. Ye , R. Vajtai , B. I. Yakobson , H. Terrones , M. Terrones , B. K. Tay , J. Lou , S. T. Pantelides , Z. Liu , W. Zhou , P. M. Ajayan , Nat. Mater. 2014, 13, 1135. 25262094 10.1038/nmat4091

